# Characterization of a fractured basement reservoir using high-resolution 3D seismic and logging datasets: A case study of the Sab’atayn Basin, Yemen

**DOI:** 10.1371/journal.pone.0206079

**Published:** 2018-10-25

**Authors:** Waleed Bawazer, Aref Lashin, Mostafa M. Kinawy

**Affiliations:** 1 Petroleum and Natural Gas Engineering Department, College of Engineering, King Saud University, Riyadh, Saudi Arabia; 2 Geology Department, Faculty of Science, Benha University, Benha, Egypt; 3 Mining and Petroleum Engineering Department, Faculty of Engineering, Al-Azhar University, Cairo, Egypt; Universite de Bourgogne, FRANCE

## Abstract

The Sab’atayn Basin is one of the most prolific Mesozoic hydrocarbon basins located in central Yemen. It has many oil producing fields including the Habban Field with oil occurrences in fractured basement rocks. A comprehensive seismic analysis of fractured basement reservoirs was performed to identify the structural pattern and mechanism of hydrocarbon entrapment and reservoir characteristics. A 3D post-stack time migration seismic cube and logging data of 20 wells were used and several 2D seismic sections were constructed and interpreted. Depth structure maps were generated for the basement reservoir and overlying formations. The top of the basement reservoir is dissected by a set of NW-SE step-like normal faults (Najd Fault System) and to a lesser extent, by secondary NNE-SSW oriented faults (Hadramauwt System). The Najd Fault System is dominant and dissects the reservoir in the middle of the field into two prospective uplifts. The northern and northeastern areas constitute the deep-seated downthrown side of the reservoir. Hydrocarbon emplacement is through the fault juxtaposition of the fractured basement against the organic shale source rock of the overlying Madbi Formation. Hydrocarbons are hosted in basement horsts formed by fault-controlled blocks and overlain by the regional seal of the Sab’atayn Formation. The basement reservoir rock is mainly composed of granite, quartz-feldspar, weathered silica, and mica minerals. Fractures were identified from the outcrops, cores, image logs, and the petrophysical analysis. Hydrocarbon saturation was observed in the upper and middle parts of the reservoir, more specifically in front of the highly fractured sections. The fracture porosity was less than 5% and the dead oil had an API gravity of 40° with no H_2_S or CO_2_. In conclusion, the structural highs of the Habban Field are of interest because most oil producing wells are drilled into them. We recommend extending the drilling and development activities in these uplifts.

## Introduction

Basement reservoirs are a subset of naturally fractured reservoirs and the term “basement” refers to crystalline formations ranging from intrusive and extrusive magmatic bodies (especially granites) to the family of low- to medium-grade metamorphic rocks [1–9]. The term basement is used for a range of intrusive or extrusive igneous and metamorphic rocks beneath an unconformity at the base of a sedimentary sequence. A large portion of the world’s oil reserves is found in naturally fractured reservoirs; fractured reservoirs contain more than 20% of the world’s remaining oil and gas resources [7,10,11].

**Fig 1:**
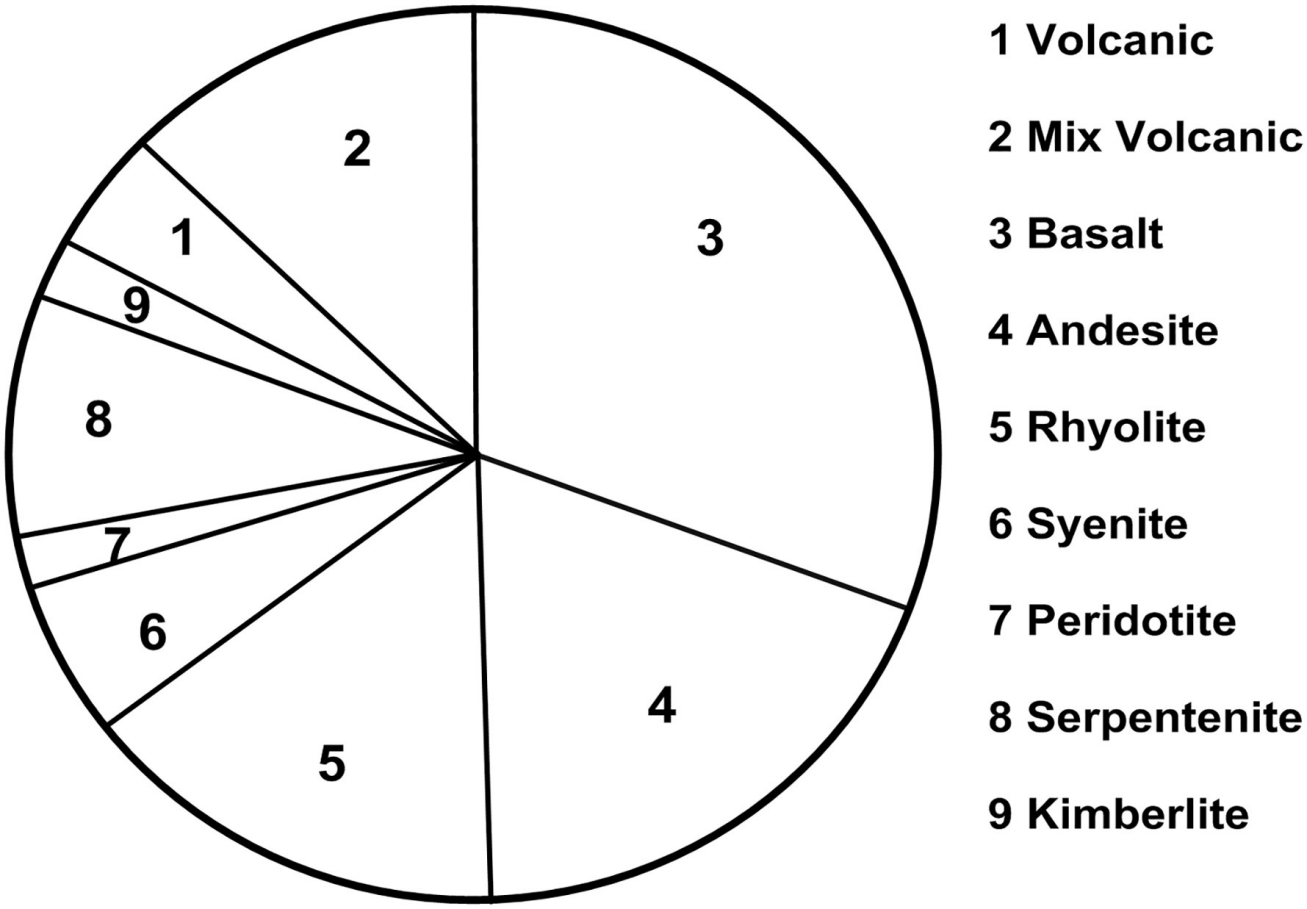
Distribution of hydrocarbons in igneous rocks after Petford and McCaffrey [12]. The figure is similar but not identical to the original image and is used for illustrative purposes only.

A global summary of different types of igneous rocks, describing hydrocarbon deposits, was prepared by Petford and McCaffrey [12] based on the review by Schutter [13] (Fig 1). The distribution of hydrocarbons in igneous rocks shows that basalts, andesites, and rhyolites constitute 75% of hydrocarbon-bearing igneous rocks. Although it is not common that hydrocarbons are retrieved from crystalline basement rocks, naturally fractured basement reservoirs have been known and exploited by the hydrocarbon industry since 1948 [14].

Fractured basement reservoirs occur in more than 25 basins in over 30 countries. The most famous is the White Tiger offshore oil field in the Cuu Long Basin in Vietnam. The reservoir was discovered in 1986 and has a cumulative production of 180 MBBL. Approximately 85% of hydrocarbon production in Vietnam is from fractured basement rocks [15,16]. The Wilmington Field in the United States was discovered in 1945 and produced 22 MBBL till now.

In the Middle East and Yemen, basement reservoirs are encountered in many locations. In Egypt, the most famous basement reservoir is the Zeit Bay Field, which was discovered in 1981 in the western margin of the southern Gulf of Suez. The production started at 20,000 BBL/D, reached its peak of 80,000 BBL/D in the mid-1980s, and declined to about 50,000 BBL/D in 2011 [17,18]. Other basement fields including Ashrafi, Hilal, and Shoab Ali Geisum were discovered in the southern Gulf of Suez [19]. In Libya, the Nafoora-Augila Field is one of the giant oil fields in the Sirte Basin. It produces oil from the basement granite with daily production rates ranging from 1200 BBL to 14,000 BBL. The earliest exploration for hydrocarbons in Yemen commenced in 1961 within the Red Sea coastal region. However, Yemen has entered the era of oil in 1984 upon the announcement of the first commercial oil discovery at the Alif Field of the Sab’atayn Basin. Approximately 50% of the hydrocarbon production comes from the basement rocks, mostly located in Masilah and Sab’atayn Basins [16]. The Habban Field is the major hydrocarbon producer from the basement reservoirs in Block S2 in the Sab’atayn Basin.

The aim of this work is to perform comprehensive seismic and petrophysical analyses of a naturally fractured basement reservoir located in Block S2 of the Habban Field in the Sab’atayn Basin to detect reservoir characteristics and identify the structural/stratigraphic elements that control the hydrocarbon entrapment using the three-dimensional (3D) seismic data and well logs [20–25].

## Fractured basement reservoirs in Yemen

**Fig 2:**
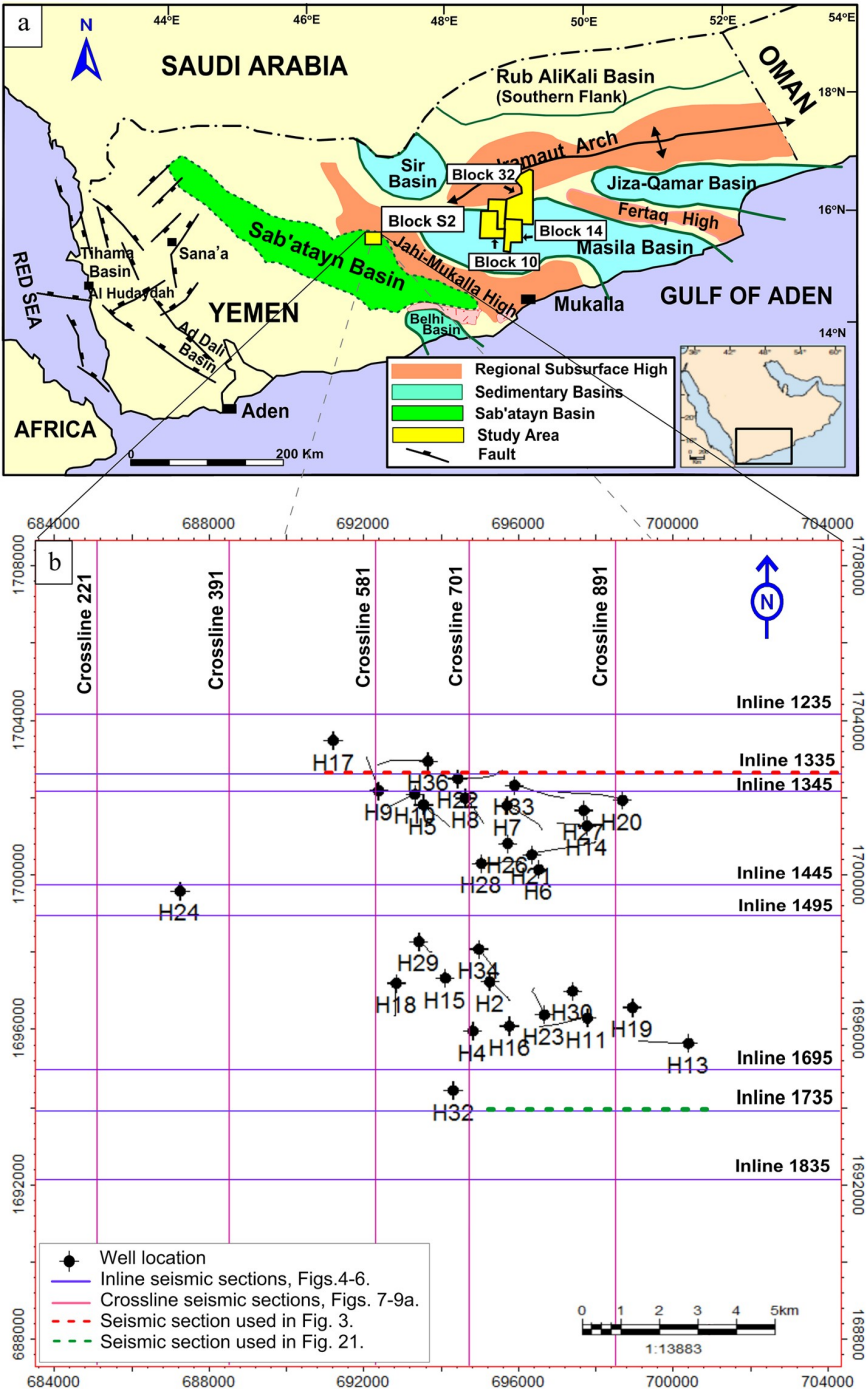
a) Geological map of Yemen showing the Sab’atayn basin (redrawn by the authors), and b) well location map of Habban field-block S2 indicating the inline and crossline seismic sections that are used in the study.

Basement rocks are among the most important targets in recent exploration efforts in Yemen. Hydrocarbons within fractured basement rocks were detected more than a decade ago in ten blocks. Currently, only five out of those ten blocks (Blocks 14, 10, 32-S, 53-S, and S2) are producing (four are located in the Masilah Basin and one in the Sab’atayn Basin) (Fig 2).

### Block 14-Masilah

A significant amount of oil has been extracted in Block 14- Masilah with the discovery of 19 fields since 1991. The main reservoirs in the block are Qishn clastics, Upper Saar clastics and carbonates, and Madbi carbonates with an oil density between 28 and 32° API in addition to light oil (41° API) in fractured basement rocks of Sunah and North Camal areas.

### Block 10-East Shabwa

Block 10 field has been producing oil since 1998 and the production has increased from 15 MBOPD to 74 MBOPD after the discovery of fractured basement reservoirs. The produced oil is light oil with 41° API from Kharir and Wadi Taribah.

### Block 32-S. Hwarim & Block 53-East Saar

Block 32 has had an average production of 2.3 MBOPD since 2005 from two main reservoirs, Qishn Clastics and the fractured basement. It is located in the north sector of the Masilah Basin and the main reservoirs are Qishn clastics, Saar sand and carbonates, and fractured basement rocks with intermediate oil density of 28–32° API, similar to Block 14.

### Block S2-Habban Field

Block S2 is located in the northern part of the Sab’atayn Basin, which is an NW-SE trending Late Jurassic intracratonic rift basin. Since the middle of 2005, the block has produced 17 MBOPD, mainly from the complex fractured basement reservoir in addition to secondary targets (Lam sand and Shuqra Formation). The produced oil is light with a density between 35 and 42° API. There is also a big gas reserve in the field.

## Geological setting of the Sab’atayn Basin

Geologically, Yemen is situated in the southwestern portion of the Arabian plate, in which the basement complex is a part of the Arabian Shield. The geology of Yemen is largely characterized and driven by two major tectonic periods. The first events took place in the Late Jurassic–Early Cretaceous when the Mesozoic rift basins developed by the breakup of Gondwana (separation of India–Madagascar from Africa–Arabia). The second period is represented by the tectonic activity related to the opening of the Gulf of Aden and the Red Sea by the separation of the Arabian plate from Africa and the collision of the Arabian Peninsula with Eurasia in the Cenozoic (i.e., Late Oligocene–Pliocene) [26].

The Sab’atayn Basin is among the most prolific Mesozoic rift basins in Yemen; it is 50 to 120 km wide and more than 450 km long (Fig 2a). From northwest to southeast, it comprises several sub-basins, including Al-Jawf, Marib, and Shabwa. It is the oldest rift basin among the Mesozoic basins and oriented in the NW-SE direction following the trend of the Precambrian Najd System fault. The study area constitutes a part of the Habban Field located to the west of Hadramauwt in Armah District (Shabwa Province) in central Yemen. The study area is between latitudes 682700 and 704920 N and longitudes 1687280 and 1708000 E (Fig 2b). It occupies an area of 387 km^2^ in Block-S2, which is mainly covered by the flat sandy desert of Ramlat Al-Sab’atayn with sand dune ridges in the north, where the Empty Quarter lies. The altitude of the block is approximately 800 m above sea level with no topographic features except the high area of Jabal AlUqlah in the east.

**Fig 3:**
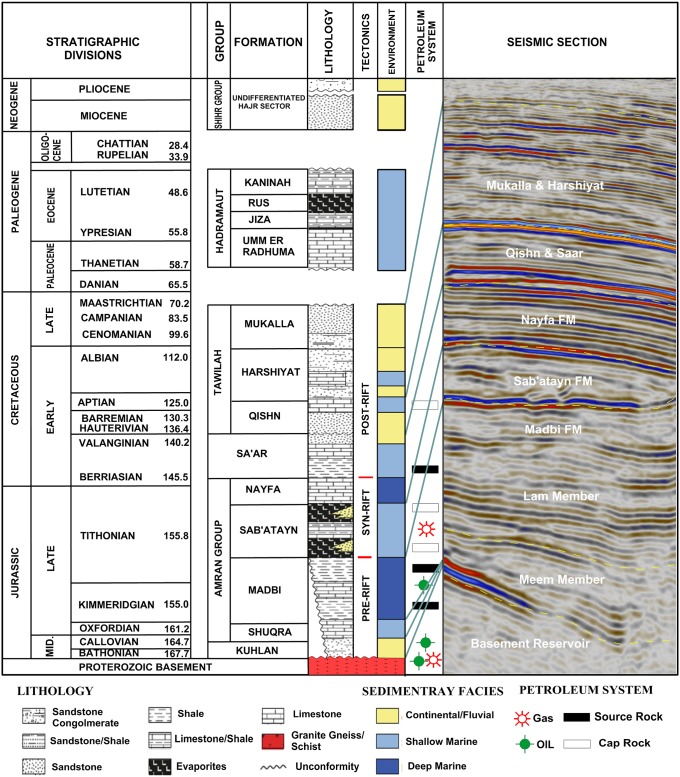
Generalized stratigraphic column of Sab’atayn Basin modified after As-Saruri and Sorkhabi and Tari et al. [27,28]. The stratigraphic units and tectonic events are following Beydoun et al. and Casto [26,29]. Stratigraphic units are correlated with seismic facies through 2D seismic section taken from the present study (middle part of inline 1735, see Fig 2b for location). The figure is similar but not identical to the original image and is used for illustrative purposes only.

The generalized stratigraphic column of Sab’atayn Basin is shown in Fig 3 [27,28]. The stratigraphy and tectonic events are following Casto and Beydoun et al. [26,29]. Stratigraphic units are correlated with seismic facies through 2D seismic section taken from inline seismic section 1735 (See Fig 2b for location). The Sab’atayan basin is dominated by a thick Mesozoic succession with an average thickness reaching up to 2500 m. It shows rock units range in age from Jurassic to Cretaceous overlie the pre-Cambrian basement rocks in addition to Quaternary exposure (Fig 3).

The stratigraphic succession of the Sab’atayn basin is can be classified into three main tectono-stratigraphic megasequences:

1. Pre-rift sequences in the basin are represented by Wajid, Akbarah, Kuhlan, and Shuqra formations. The Kuhlan Formation mainly includes fluviatile and arkosic clastic rocks [29]. These continental rocks are conformably overlain by shallow-marine fossiliferous carbonates of the Shuqra Formation (Fig 3).
2. Syn-rift sequences are characterized by horsts and nested fault blocks that developed during Late Jurassic and Early Cretaceous period [30] and include Madbi, Sab’atayn, and Nayfa formations. The Madbi Formation is divided into two members: the lower Meem and the upper Lam [29]. The lower Meem Member consists of clastic turbidities and shales that are important source rocks, whereas the upper Lam Member is mostly composed of laminated organic-rich shales and considered one of the most prolific oil-prone source rocks in the basin [31,32]. During Late Syn-rift, the continental sediments of the Sab’atayn Formation were deposited in the grabens from the north end of the western basin along rift margins. It consists of a thick sequence of clastic and evaporite sediments and offers good regional seal for the underlying hydrocarbon reservoirs. During the Late Jurassic–Early Cretaceous, the rift system was active and accompanied by the accumulation of carbonates of the Nayfa Formation in a shallow-marine shelf environment.
3. Post-rift sequences are represented by earliest Lower Cretaceous to Upper Cretaceous deposits and comprise the Saar, Qishn, and Tawilah formations. The Saar Formation is mainly composed of limestone and dolomite, with mudstone and sandstone intercalations [28]. The Qishn deposits represent a transgressive marine sequence progressing from east to west across Yemen that grade laterally westward into the clastics of the non-petroleum-bearing Tawilah Formation.

## Data and methodology

A cube of 3D post-stack time migrated seismic data (PSTM) and logs of 20 wells were used to infer the structural setting, entrapment style, and characteristics of fractured basement reservoirs in the Habban Field.

### Seismic data analysis

The seismic data used in this study are represented by a 3D seismic cube forming a seismic grid of 20 km × 20 km. The Petroleum Exploration and Production Authority (PEPA) of Yemen gave permission to OMV Company to conduct the survey on this site. The data quality is good and has been collected since 2007 by OMV Company after oil was discovered in two wells. These two wells were drilled in 2005 based on an old 2D seismic survey, which was mainly acquired by OXY Company (1126 line km), Preussag (351 line km), and OMV (130 line km). The study area had been covered by 2D seismic lines data since the 1990s. The 3D seismic cube is represented mainly by PSTM that covers an area of 387 km^2^ between latitudes 682700 and 704920 N and longitudes 1687280 and 1708000 E (Fig 2b).

#### Data processing & time-to-depth conversion

The data were processed to remove noise that could affect the imaging algorithms and mask the real data. The Ricker type wavelet has been used and the unwanted coherent energy and multiples were removed. Extended White method has been used for wavelet extraction due to its greater viability with the studied dataset [33]. Since the study area is 800 m above sea level, time shifts caused by topography and near-surface velocity anomalies were also considered. The difference in lithology between the basement reservoir and the overlying sediment rocks has also been taken into consideration.

Time-to-depth conversion is an important step and is based on a specific velocity model. The average velocity model (stacking velocity) as matched with the data from existing well tops was mainly used in this study to obtain a precise picture of geological surfaces. Geostatistical techniques were used for data integration to achieve the depth conversion. The uncertainties in the depth prognosis were +/- 50 m for post-Sab’atayn formations and +/- 70 m for pre-Sab’atayn formations.

#### Horizon/Fault picking and mapping

In the Habban Field, seismic-to-seismic and seismic-to-well tie analyses were performed to detect the horizons of interest. Five main horizons were chosen and mapped, Qishn, Nayfa, Sab’atayn halite, and Madbi formations, as well as the basement reservoir. Control points were selected using the formation tops taken from the well records. Faults are special surfaces whose traces are displayed on structural maps and form conduits for reservoirs. Different fault systems were also picked in crosslines and inlines and mapped. Finally, a depth structure map was constructed for each picked horizon by marking the top of the selected horizon over the extended 3D seismic survey. These maps are conventionally used to interpret the prevailing structural trends and identify the prospective leads.

### Petrophysical analysis

Complete well logs of 20 wells in the Habban Field were used in petrophysical analysis. These data were provided in digital Log ASCII Standard format (LAS files). They include neutron logs, dipole sonic data, density data, gamma-ray (natural & spectral), resistivity logs, image logs, photoelectric factor, and caliper logs. The data have a vertical resolution of 0.15 m. Interactive Petrophysics software (IP v.4.10) was used to represent different curves, extract well headers, and execute final interpretations. A comprehensive petrophysical analysis was carried out over the basement reservoir rocks using several qualitative and quantitative well logging procedures [18,20,34–36]. The analysis was performed to characterize the crystalline basement reservoirs by determining their lithology, porosity, and fluid content. The available core and rock cutting analyses of the Habban-1 Well were used for fracture/lithology description. A brief interpretation of the lithology of the overlying source (Madbi Formation) and seal rocks (Sab’atayn Formation) is provided in Section 4.

Neutron-density combinations (N-D) are mainly used for delineating the composition of the basement reservoir by the relative occurrence of different data points to the position of common basement minerals (granite point GP, quartz-feldspar point QFP, and muscovite-biotite point MB) [35,37]. A compiled plot integrating the N-D basement points and standard lithological lines for major sedimentary rocks (SWS, Neutron-Density cross-plot, CP-1e [35]) was constructed to characterize the lithological components of the basement reservoir, as well as the lithology of the overlying source rock (Lam and Meem members).

Special petrophysical models based on shallow and deep resistivity logs, photoelectric cross-sections, and spectrometry gamma-ray logs (Th, U, and K) were used for lithology and fracture porosity determination. A plot of the deep/shallow ratio (RD/RS) versus deep resistivity (RD) logs was constructed for fracture porosity estimation. The plot is usually used to discriminate between fractured, un-fractured, and hydrocarbon-bearing fracture zones [20,38,39]. The borehole image logs were also employed for fracture system identification. They provide an electronic image by measuring the resistivity variation of the fluids and rocks as well as the structural features crossing the borehole. Schlumberger’s Formation Micro-Imager (FMI) logs with a vertical resolution of 0.20 inches (5 mm) were employed in this study. The results from the FMI analysis and those from seismic data are compared (direction and dip of fractures) through rose diagrams.

## Interpretation of seismic data

A 3D post-stack seismic cube was analyzed to study the general structural features of the Habban Field in the Sab’atayn Basin. Several 2D interpreted seismic sections (inline and crossline) were constructed. In addition, detailed subsurface structural mapping was generated for the tops of the Qishn, Nayfa, Sab’atayn, and Madbi formations, as well as the basement reservoir.

### 2D seismic sections

Since seismic data are in the form of 3D seismic cubes, large numbers of 2D inline and crossline seismic sections are available for analysis and interpretation. The inline sections run from east to west and aligned along the north-south direction from shot point 1005 (N) to 2025 (S), whereas the crossline sections run from north to south and aligned along the W-E direction from shot point 101 (W) to 1201(E).

#### Inline seismic sections

**Fig 4:**
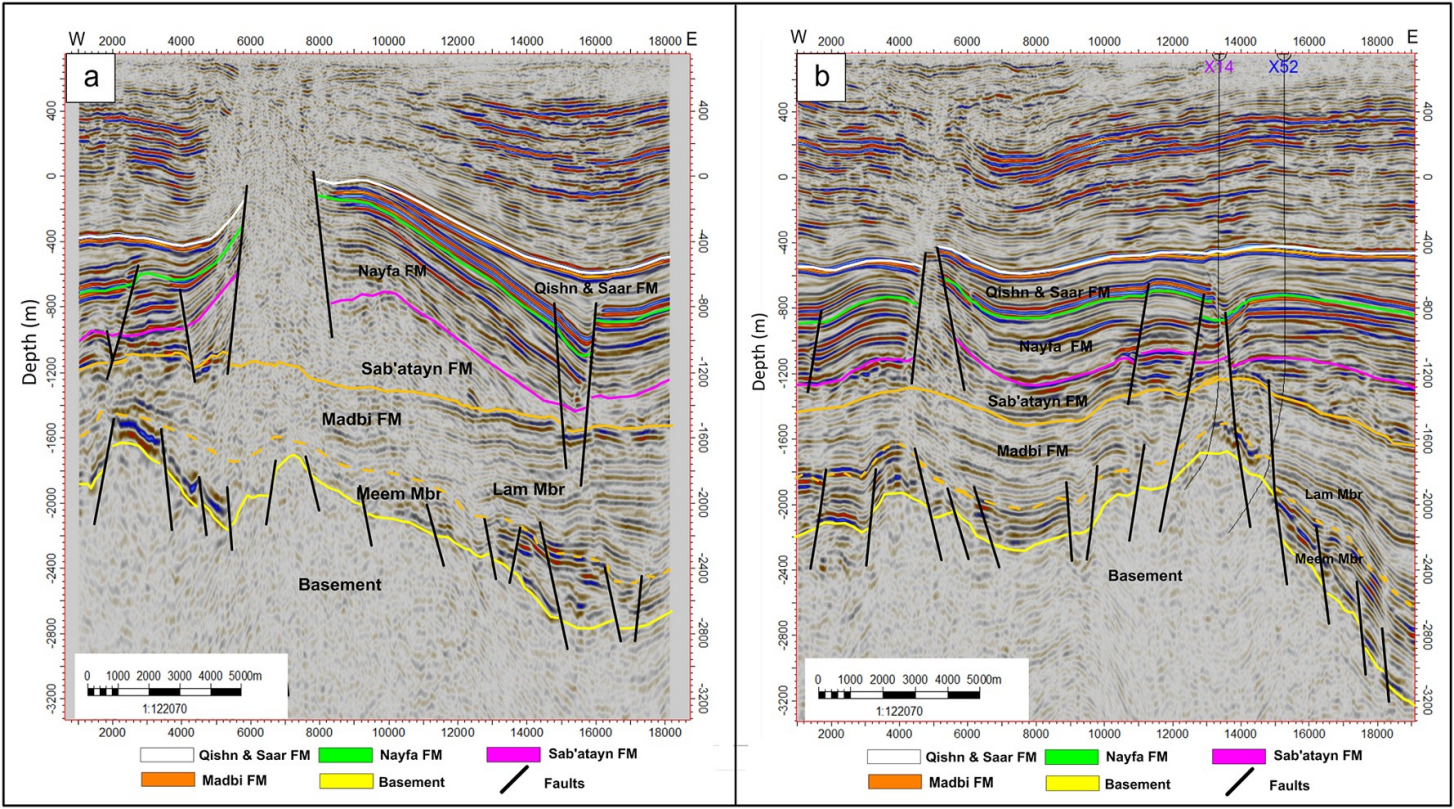
a) 2D interpreted inline seismic section 1235, and b) 2D interpreted inline seismic section 1345. The intrusion of the Sab’atayn salt is well seen in the western side of inline section 1235 associated with basement reservoir uplift.

**Fig 5:**
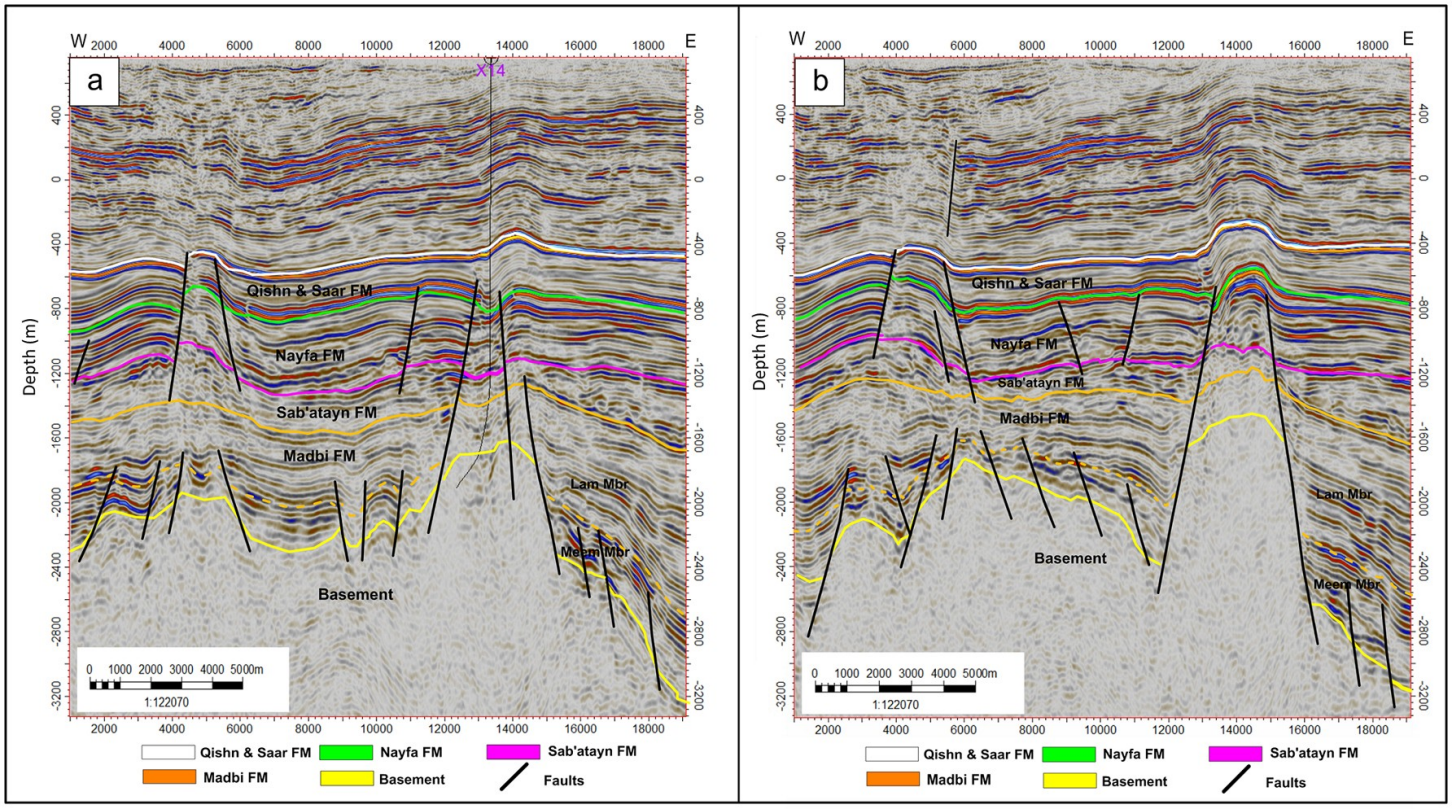
a) 2D interpreted inline seismic section 1445, and b) 2D interpreted inline seismic section 1495. The uplift of Habban North Horst (HNH) is well developed in the right side of inline section 1495. The uplift bounding faults die at the top of Nayfa Formation.

**Fig 6:**
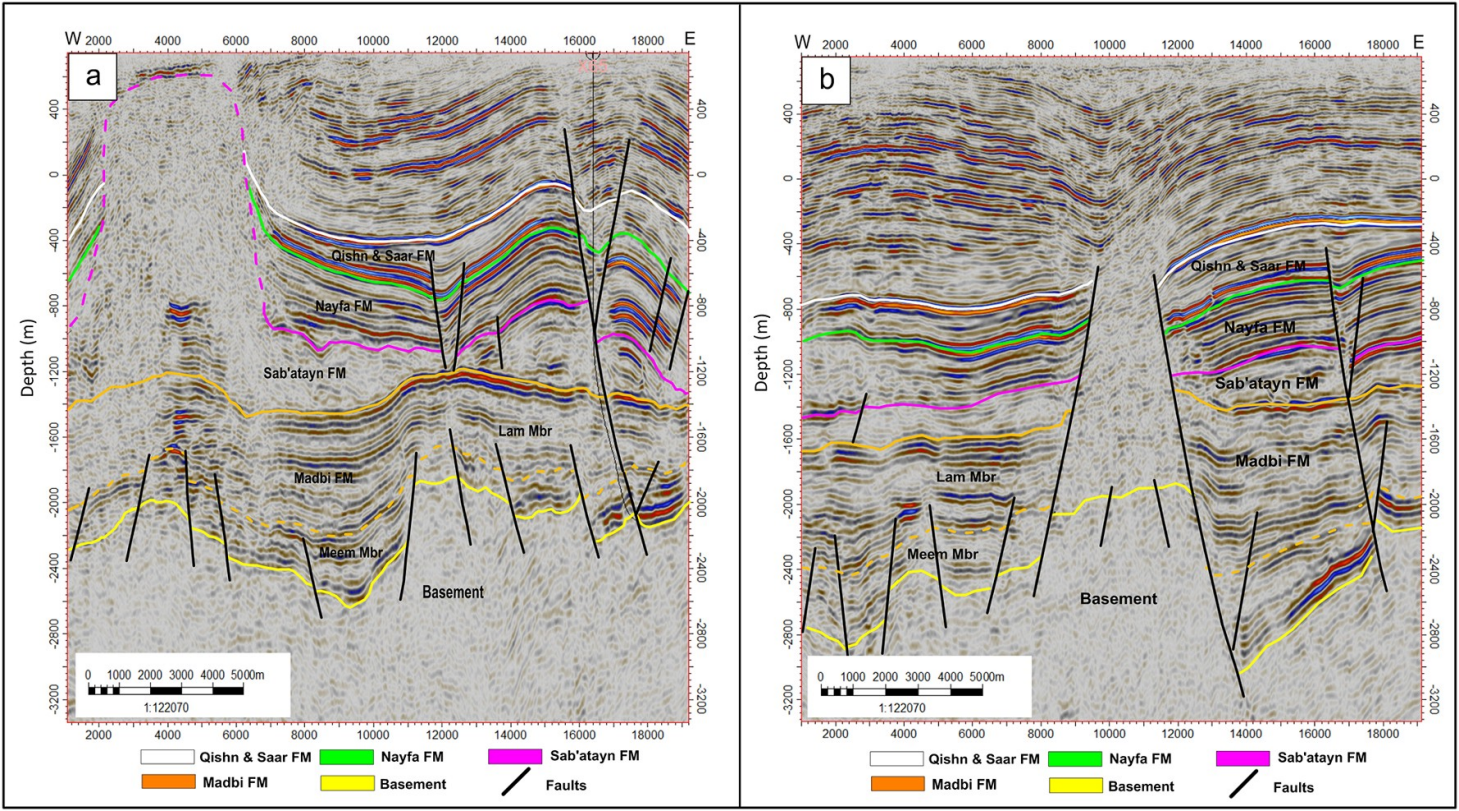
a) 2D interpreted inline seismic section 1695, and b) 2D interpreted inline seismic section 1835. The uplift of (Habban Central Horst, HCH) is well developed in the middle of inline section 1835. The uplift bounding faults reach the top of Qishn Formation. A typical cylindrical intrusion of the Sab’atayn diaper reaches the near surface shallower strata left of inline section 1695.

Figs 4(a) and 4(b)–6(a) and 6(b) show six E-W interpreted inline seismic sections (Inlines 1235, 1345, 1445, 495, 1695, and 1835) selected to cover the study area from north to south. The top of the basement reservoir is affected by a large number of normal faults, mostly along the NW-SE direction (Jurassic rift faults that follow the Pre-Cambrian Najd Fault system) [30]. A few faults are aligned along the NNE-SSW direction (Gulf of Aden fault system/Hadramauwt fault trend). Most of these faults affect the top of the basement rocks for a few hundred meters and extend upward to die out on the Lam Member of the overlying Madbi Formation. For this reason the majority of the drilled/production wells in the study area is designed to reach these few hundred of meters at most and in most cases is inclined to catch as much as of the reservoir fractures/fault system as possible. Some small-scale horsts and grabens were present in the western part of the study area (the left side of seismic sections 1235, 1345, and 1495 in Figs 4a, 4b and 5b, respectively). However, a strong structural uplift (Habban North Horst, HNH) developed in the northern middle part of the study area, where two major normal faults divide the top of the basement reservoir and overlying succession until the bottom of the Qishn Formation (the area between shot points 12000 and 16000 in seismic sections 1345, 1495 in Fig 5a and 5b). A similar promised basement uplift (Habban Central Horst, HCH) developed in the southern middle part of the study area and is represented by strong cutting faults that divide the whole section, tapping the top of the Qishn Formation with a big heave (the seismic section 1835 in Fig 6b). These structural uplifts are originally related to old reliefs that are re-worked during the Jurassic rift and the Gulf of Aden /Hadramauwt fault systems [30]. The top of the basement reservoir between HNH and HCH is normally dissected by low magnitude faults (the seismic section 1695 in Fig 6a). Throughout the study area, the top of the basement reservoir is strongly affected by a group of high-angle step-like normal faults with a big throw resulting in deeper blocks to the east and northeast of the Habban Field (the right side of inline sections, 1235, 1345, and 1495 in Figs 4a, 4b, and 5b).

Aside from the major uplifting faults, the structural elements affecting the overburden stratigraphy (formations overlying the basement reservoir, i.e., Madbi, Sab’atayn, Nayfa, and Qishn formations) can be categorized into two main groups. The first group is represented by low-magnitude normal faults that extend from the underlying basement reservoir to affect the lower part of the Madbi Formation (Meem Member). These faults die out in the Lam Member and never extend to higher overlying formations. The second group is represented by the faults that cut through Qishn, Nayfa, and Sab’atayn formations. Some of these faults detach in the Sab’atayn Formation and others extend to offset the uppermost Lam Member.

The intrusion of the Sab’atayn salts into the overlying layers usually follows the weakness zones and is greatly facilitated in faulted areas, forming thick salt diapirs. This phenomenon typically occurs in the highly-faulted low-pressure parts at the northwestern and middle-western of the study area (the left sides of seismic sections 1235 and 1695 in Figs 4a and 6a, respectively).

#### Crossline seismic sections

**Fig 7:**
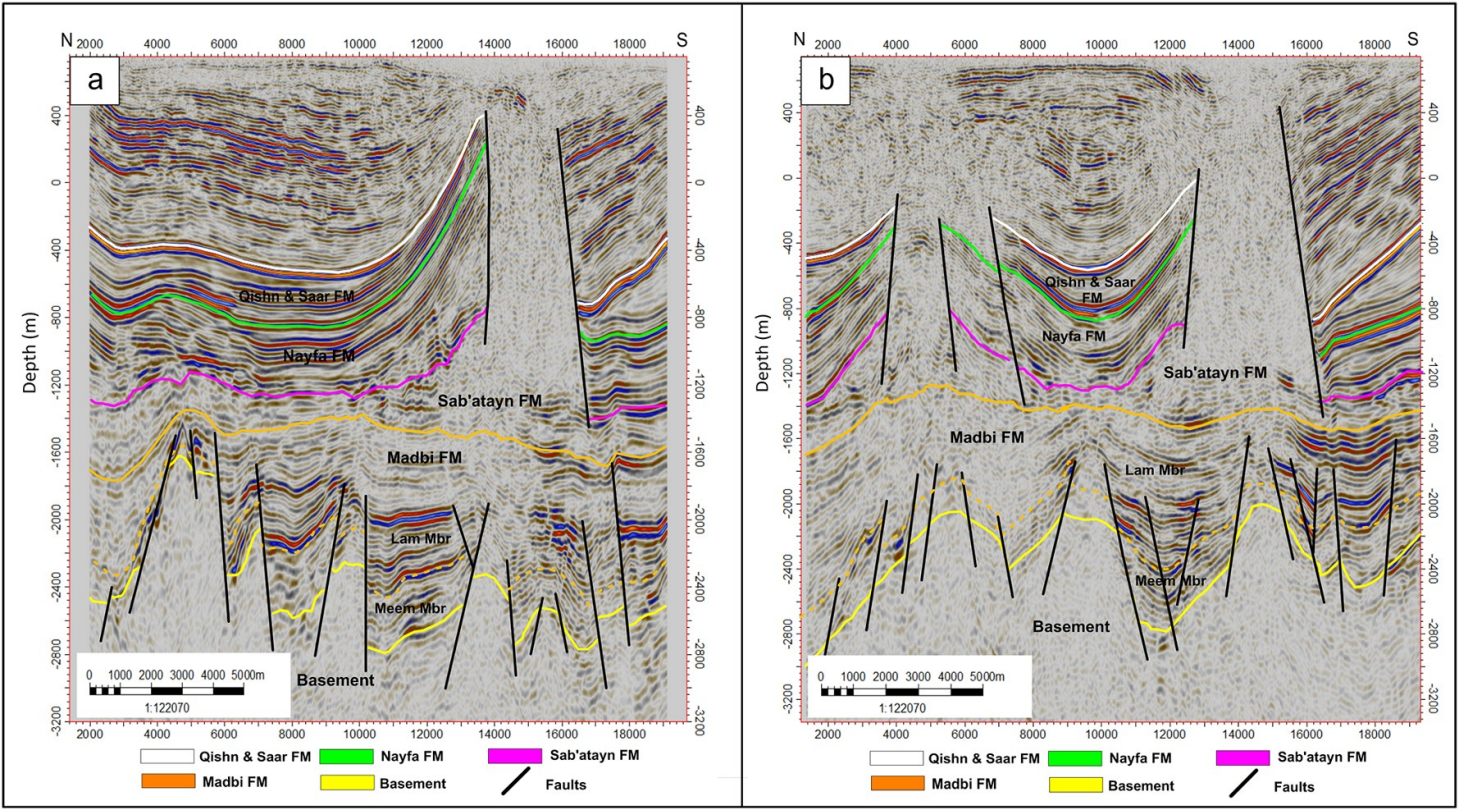
a) 2D interpreted crossline seismic section 221, and b) 2D interpreted crossline seismic section 391. The intrusion of the Sab’atayn salt is well seen in both sections. The faulted-basement reservoir blocks and parts of the overlying Madbi Formation are juxtaposed along fault plans.

**Fig 8:**
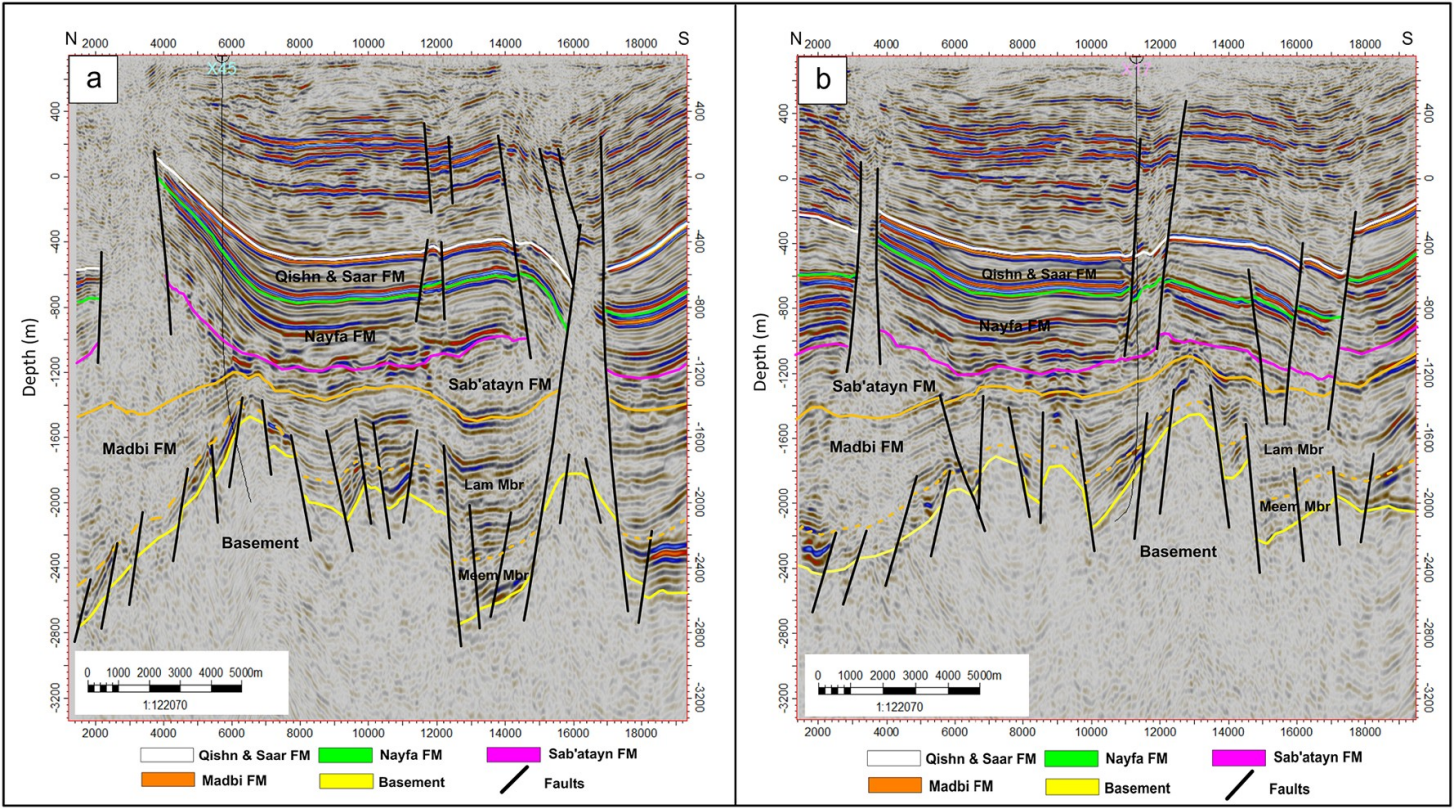
a) 2D interpreted crossline seismic section 581, and b) 2D interpreted crossline seismic section 701. One strong uplift is indicated in the right side of crossline section 581. Most of the faults effecting the basement reservoir die out at the Lam Member of Madbi Formation. Extensive faulting is indicated in the overlying section of the Late Cretaceous, undifferentiated Palegone and Neogene sediments (see stratigraphic column at Fig 3).

**Fig 9:**
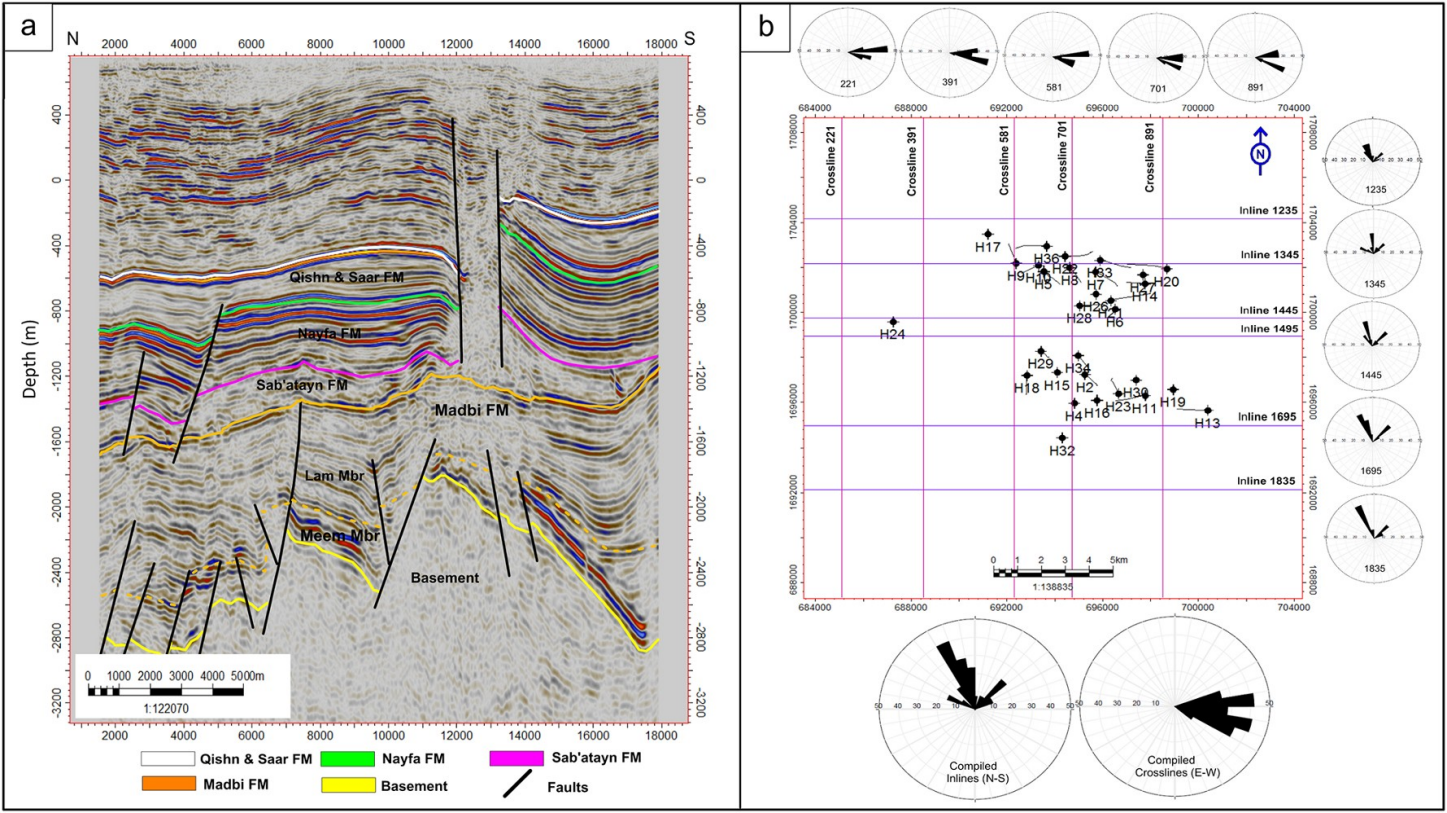
a) 2D interpreted crossline seismic section 891, and b) rose diagram showing the fault system analysis along the N-S and E-W directions.

Five interpreted crossline seismic sections (crosslines 221, 391, 581, 701, and 891) cut the study area from north to south (Figs 7–9a). The top of the basement reservoir is dissected by the step-like normal Najd fault system, which affects the lower part of the overlying Madbi Formation. The NNE-SSW Aden faults are also detected. Some V-shaped graben structures are observed at the intersection of Najd and Aden fault systems (middle of sections 221 and 891 in Figs 7a and 9a). The top of the basement is structurally tilted in the north direction making the reservoir deeper (the west side of sections 391, 581, and 891 in Figs 7b, 8a, and 9a). This deep-thrown side of the reservoir constitutes a part of the major fault down-throw observed in the northern and northeastern parts of the field. Some uplifted basement horsts are also observed in the middle of the Habban Field (section 581 in Fig 8a). The structural faults affecting the overlying Madbi, Sab’atayn, Nayfa, and Qishn formations are separated and they are much simpler than those affecting the underlying basement. Both NW-SE and NNE-SSW trends cut through the tops of Sab’atayn and Nayfa formations.

These fault systems are mainly related to the separation between India-Madagascar plate and the African-Arabian margin (Gondwana breakup) during the Late Jurassic–Early Cretaceous. They induced the reactivation of the inherited Najd Fault system (NW-SE) and development of major Mesozoic basins in Yemen (including the Sab’atayn Basin). The opening of the Gulf of Aden was the latest major tectonic event that occurred in the study area resulting in the development of NNE-SSW fractures and faults [16,40–42]. These two major fault trends reflect two major distinct tectonic phases that affect the whole succession in the study area.

The N-S and E-W evolution of these two fault trends throughout the study area is investigated by constructing several rose diagrams for each interpreted seismic section (Fig 9b). The N-S aligned rose diagrams generated for the inline sections show that the NW-SE fault trend is structurally more developed in the southern part of the study area (area covered with inline seismic sections 1695 and 1835), whereas the NNE-SSW faults are more developed in the central and southern parts of the study area.

### Depth structural maps

**Fig 10:**
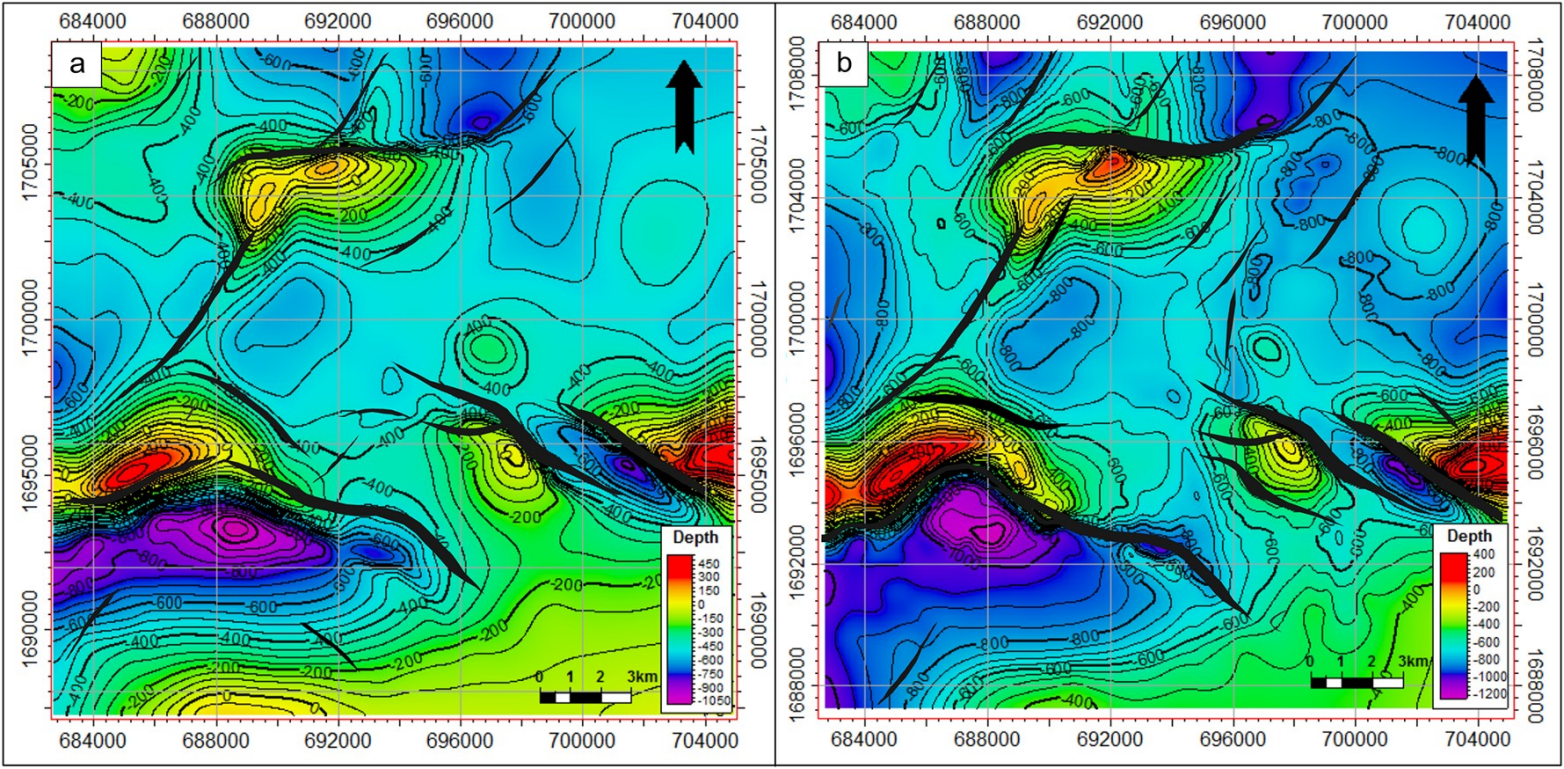
a) Depth structural map to top of the Qishn Formation, and b) depth structural map to top of the Nayfa Formation.

**Fig 11:**
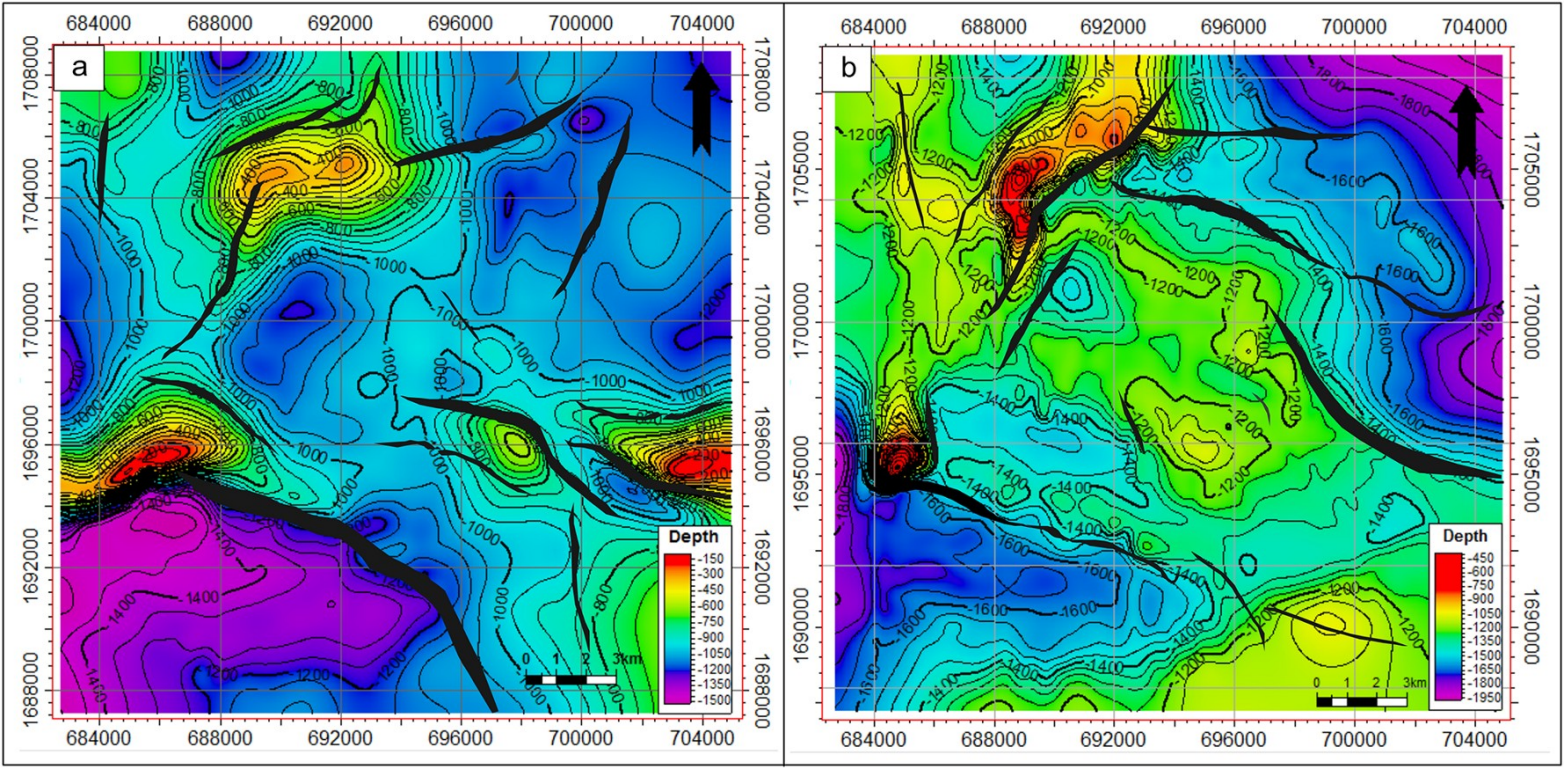
a) Depth structural map to top of the Sab’atayn Formation, and b) depth structural map to top of the Madbi Formation.

**Fig 12:**
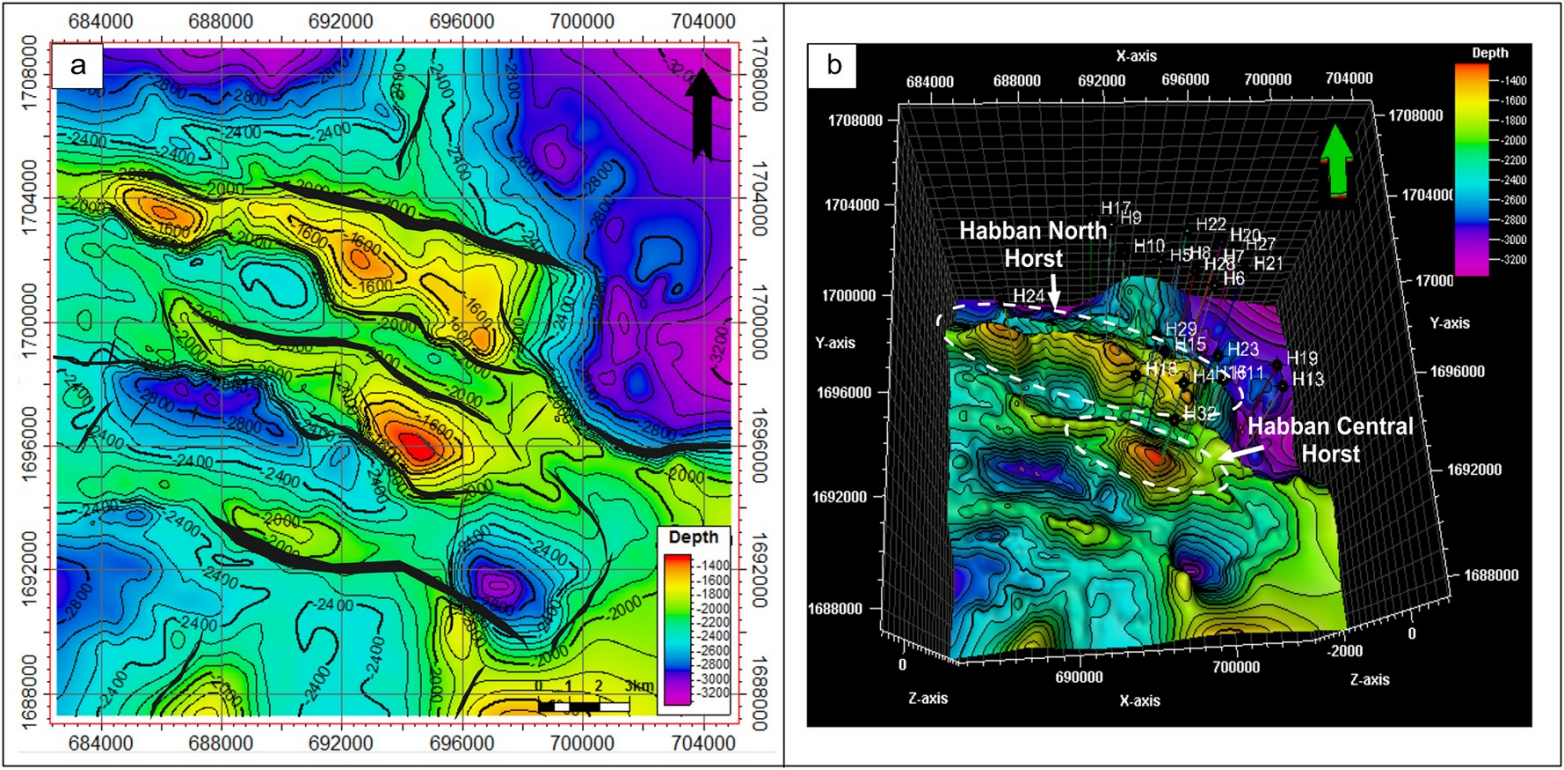
a) Depth structural map to top of the basement reservoir and b) 3D map to the top of the basement reservoir. The uplifts of the Habban North Horst (HNH) and Habban Central Horst are indicated.

Five seismic horizons, including the top of the basement reservoir, were mapped and interpreted. These horizons represent the reservoir rock and overlying petroleum system elements (Figs 10–12). Fig 10a shows the depth structure map of the top of the Qishn Formation. Qishn is a reservoir in the Masilah Basin; however, because of the halite effective seal, it is not in the Sab’atayn Basin. The top of the Qishn Formation ranges from 440 m above sea level to 1050 m below sea level. It is dissected by several faults, mostly in the NW-SE in direction, forming structural horsts and grabens. Some minor NNE-SSE and E-W faults were detected in the northern part of the study area.

The depth structure map of the Nayfa Formation is shown in Fig 10b. The Nayfa Formation is underlying the Qishn Formation and is marked at the top by a thin bed of anhydrite followed by a layer of thick limestone and calcareous claystone. It has nearly the same structural features as the Qishn Formation. The depth ranges from 400 m above sea level to 1200 m below sea level. The Sab’atayn Formation offers a regional seal of the whole petroleum system of the Sab’atayn Basin where no reservoirs for post-salt formations are detected (Fig 11a). It consists of a thick halite layer that overlies the Madbi Formation. Fig 11b illustrates the depth structure map of the top of the Madbi Formation. The surface of the Madbi Formation dips from the moderate-depth middle area to the NE and SW directions (low areas) with a general depth range of 450–1950 m. Since most of the underlying faults are low-magnitude and cut through the basement reservoir and the lower part of the Madbi Formation (die out in the Lam Member), the NW-SE normal faults were mapped with less intensity. The NE-SW fault system is detected in the north and northwestern part of the study area associated with a good structural uplift.

The top of the basement reservoir map is presented in Fig 12a. The NW-SE fault systems are more developed than the NE-SW ones, especially in the middle area where the two prospective basement horst blocks (uplifts of HNH and HCH) are separated by a deep half graben created during the late Jurassic rifting (see stratigraphic column in Fig 3; OMV, 2005 [43]). The Najd fault trend is the dominant fault system, whereas the Hadramauwt fault trend is a secondary one, which is offset in several places by the Najd faults. The HNH and HCH uplifts are of interest for further drilling and development activities. Most oil producing wells are drilled through these two uplifts (Fig 12b); however, another uplift with good potential was detected in the southeastern part of the study area.

## Petrophysical analysis & reservoir characteristics

Unlike the sedimentary reservoirs, the basement reservoirs include many lithological components (granite, quartzite or gneiss, amphibolite, and epidote-quartz breccia, etc.) and in most cases are subject to weathering and alteration processes. So, this type of reservoirs is mostly heterogeneous in composition. Typical granite consists of 10–30% quartz accompanied by alkali feldspars (sodium and potassium varieties) and mica. Ferromagnesian minerals such as hornblende and accessory minerals such as zircon may also be present. Complex mineralogical changes may occur if the basement is exposed to chemical and thermal alteration [35].

### Lithological content

**Fig 13:**
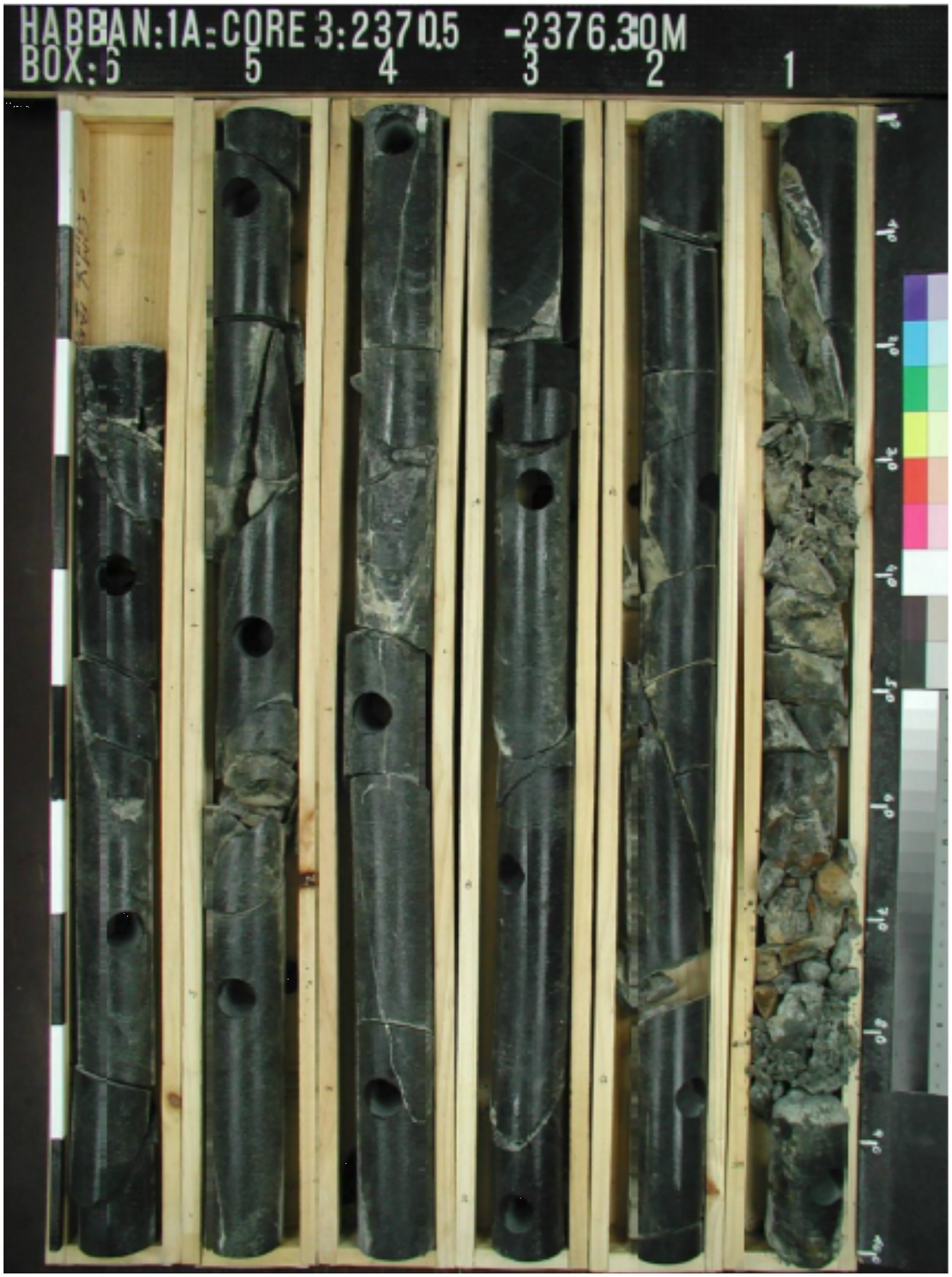
The cored basement reservoir interval 2370.5–2376.3 m at Habban-1 well. Good fracture system is indicated at the samples 1, 4 and 5. Sample 1 is completely crushed.

The available core sample from the basement reservoir interval of 2370.5–2376.3 m at the Habban-1 Well is shown in Fig 13. The basement reservoir has highly variable crystalline lithology, i.e., granite, quartzite/gneiss, amphibolite, epidote-quartz breccias, and volcanic rocks/soapstone, with low matrix porosity and permeability [28,44].

**Fig 14:**
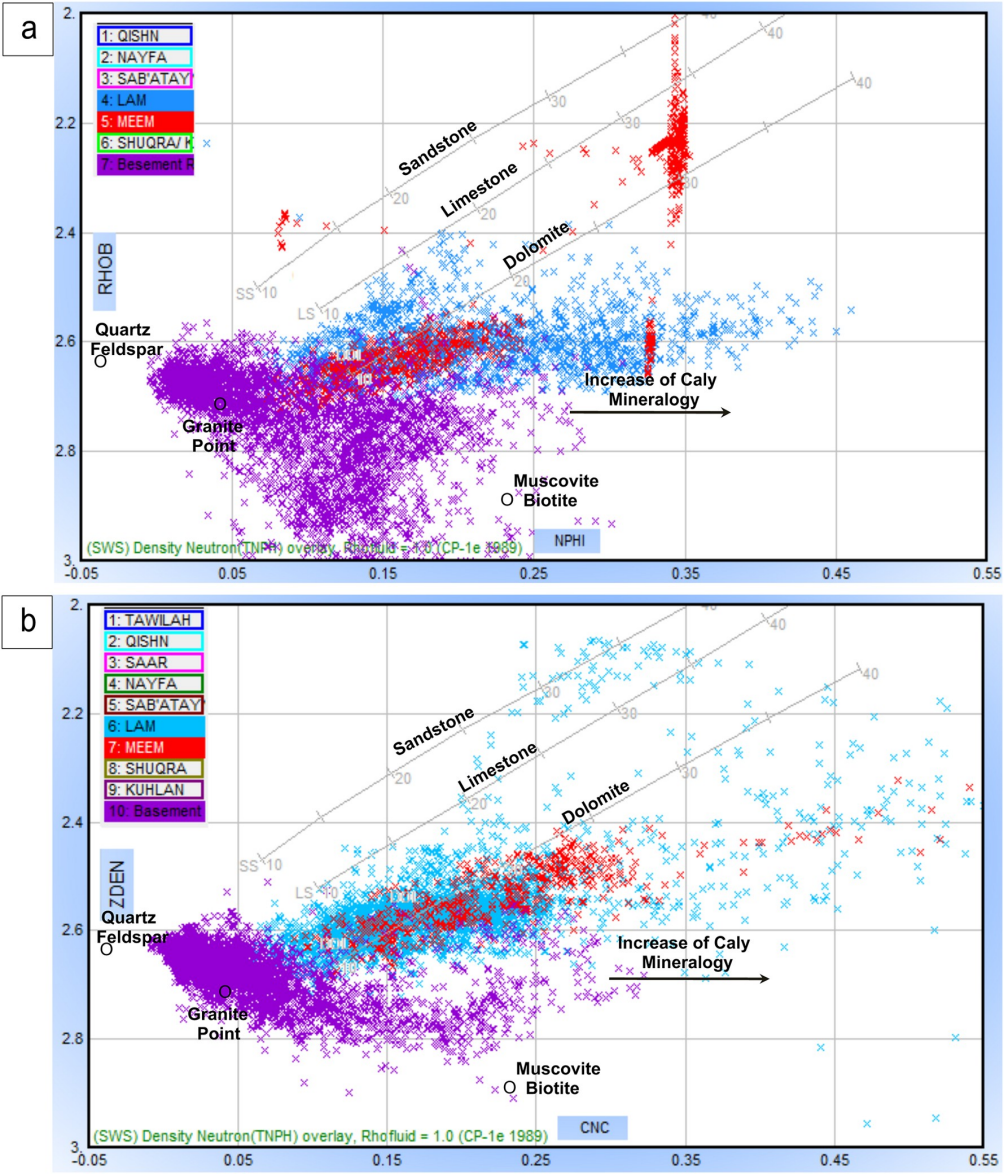
The compiled neutron-density crossplots of a) Habban-26 well and b) Habban-2A well. The datapoints of the Lam (blue) and the Meem (red) Members and the basement reservoir (violet) are presented.

Fig 14a and 14b shows the neutron-density cross-plots of Habban-26 and Habban-2A wells. Most data points at the Habban-26 Well are clustered along the GP and shifted towards the QFP, indicating the presence of granite, quantize/gneiss, and quartz-feldspar. The weathered silica and mica minerals are also present (muscovite and biotite). The downward shift of clusters in the order of ascending density indicates that some heavy and ferromagnesian minerals (hornblende and zircon) are present. The same lithological components are also indicated in the neutron-density cross-plot of the Habban-2A Well (Fig 14b). However, the percentage of quartz and alkali feldspar is higher, while the percentage of heavy and ferromagnesian minerals is low. The lithology of the overlying source rocks of the Lam and Meem members is mainly calcareous in content because most data points are between limestone and dolomite lithology lines. The Meem Member is more dense and dolomitic in composition (red points clustered close to the dolomite line). The high clay content is indicated by the data points shifted to the right below the dolomite line (Fig 14a and 14b).

### Fracture/Fault system identification

In crystalline basement reservoirs, the nature and evolution of the fracture systems have a direct influence on the fluid flow pathways. Fractures form the main conduits for fluid transmission and provide an important storage volume for basement reservoirs. They can be classified into two groups, (1) primary fractures that develop during emplacement, cooling, and decompression of magma-like joints, diaclases or aplitic/ pegmatitic dykes, and (2) secondary fault-related fractures [45,46].

#### Fracture signatures from outcrops

**Fig 15:**
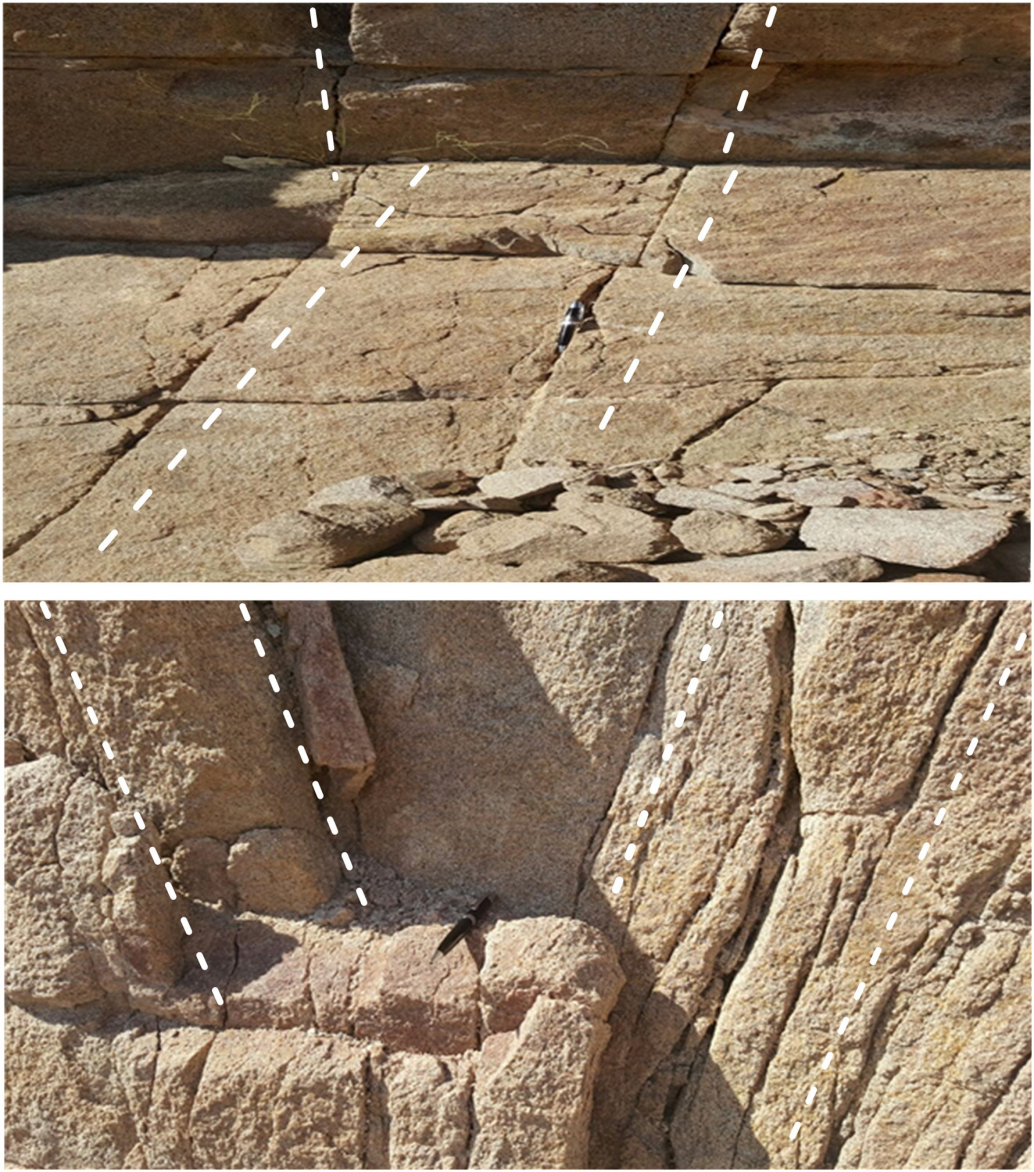
(a& b). Fracture system at exposed basement rocks at Brum area west of Mukalla, Sab’atayn Basin, Yemen. The pen denotes to the north direction. The white dashed lines indicated fracture directions.

When using fractured outcrop analogues, we should consider the influence of weathering and erosion on the original fracture network and the outcrop fracture network representative of the subsurface reservoir [47–49]. In the Mukalla-Sab’atayn Basin, the prevailing subsurface structure system (as detected from seismic data) and its influence on the surface is identified on the outcrops from the lineaments and exposed facture system. On the macro scale (from seismic to surface), the Mesozoic NW-SE structures are easy to observe at the surface, as they outline the horst and graben structure of the Sab’atayn Basin, especially in the western parts. Fig 15(a) and 15(b) shows the basement rocks exposed on the surface area of Brum to the west of the Mukalla-Sab’atayn Basin. The outcrop shows nicely oriented joint/facture system that provides, in many cases, a signature for the subsurface fracture system. Most of the joint/fracture system (width and intensity) is aligned to the north, northeast, and northwest directions.

#### Core samples & well logs

**Fig 16:**
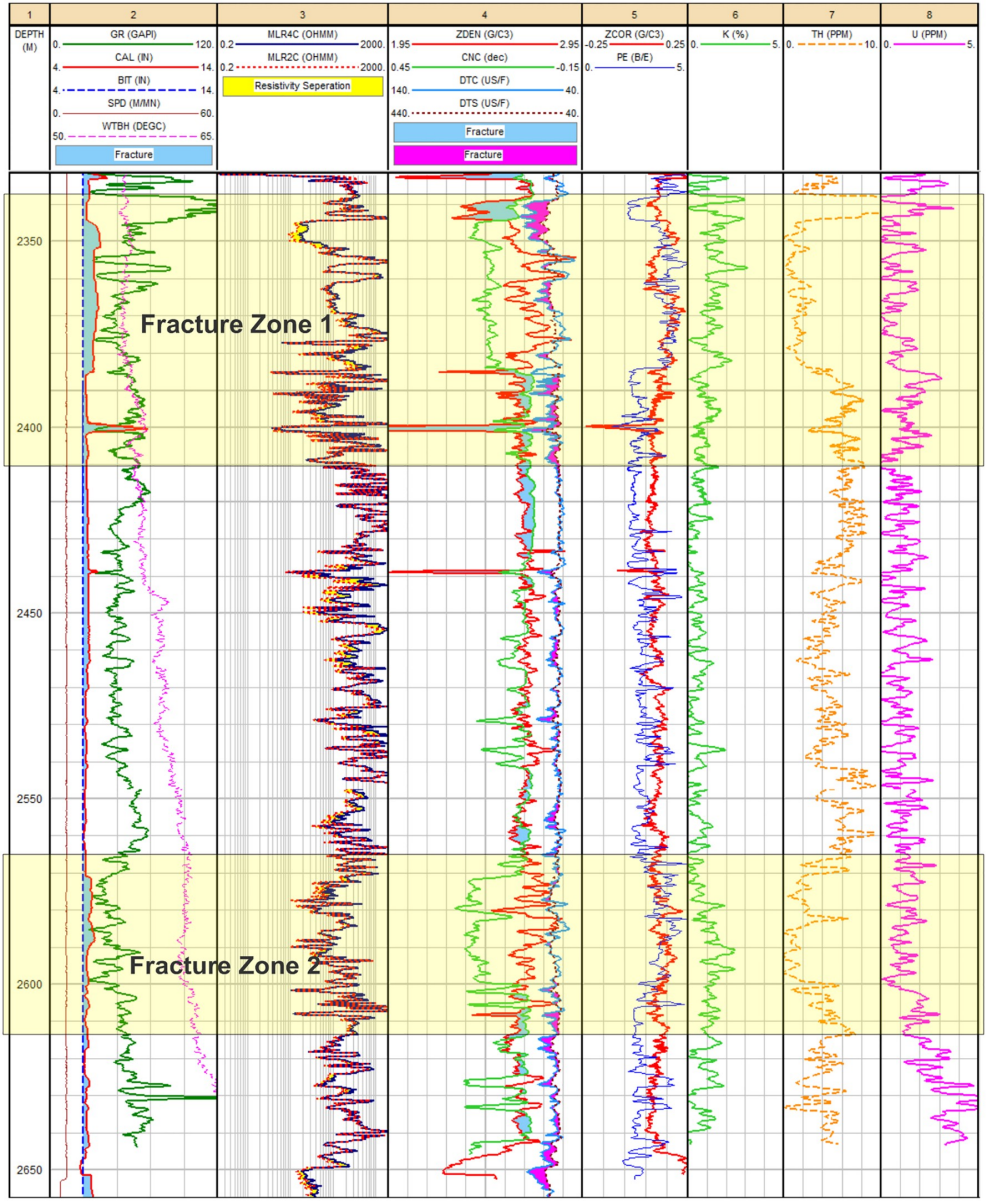
(a& b). Fracture system identification from the response of well logs at Habban-28 well.

The available core sample of the Habban-1A Well shows many fractures with different orientations (Fig 13). The NW-SE and NE-SW fracture directions that correspond to various brittle deformation episodes are clearly indicated [28,44]. Fig 16 shows the fracture system identification using an integrated logging suite that covers the basement reservoir down to a depth of 300 m at the Habban-28 Well. A significant fracture system (fracture zone 1) is detected at the depth interval of 2340–2410 m and another at a deeper zone (2565–2615 m, fracture zone 2). For the first zone, the caliper log fluctuates around the standard bit size line, indicating the increase in borehole diameter associated with an increase in gamma-ray log (Track 1). The neutron-density curve overlays also provide a good fracture signature by a decrease in the density log and an abrupt increase in the neutron log. The sonic compressional and shear interval transit times (DTC and DTS) exhibit the same curve separation (Track 4). In Track 5, the density log correction curve (ZCOR) reads higher values up to 0.20 gm/cc that indicate high photoelectric absorption factors and high potassium/uranium contents (Tracks 6, 7, and 8). The second zone shows nearly the same log characteristics.

**Fig 17:**
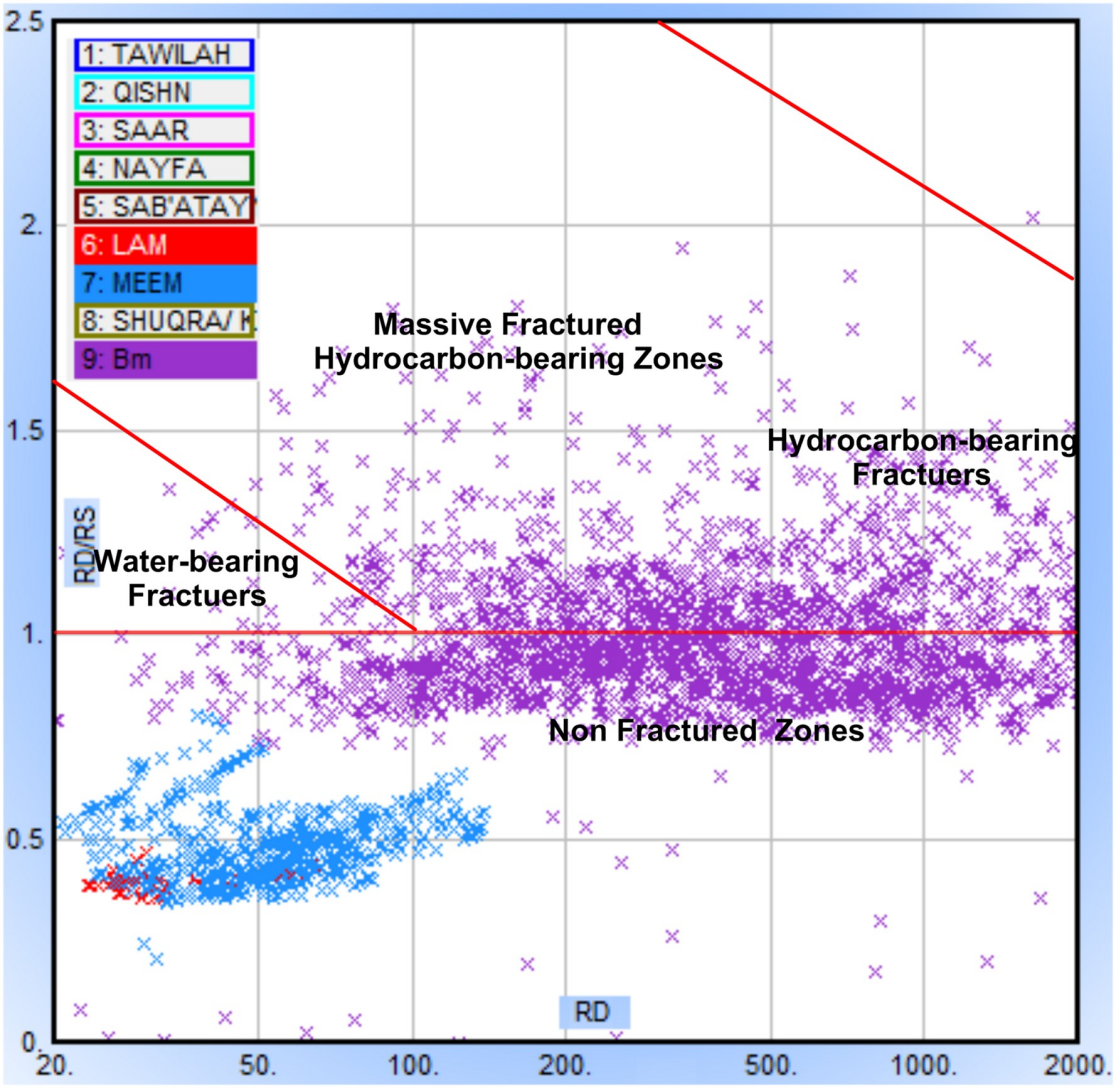
The resistivity ratio method (RD-RD/RS) for fracture system identification, at Habban-18 well. The Lam (blue) and Meem Members are located in the non-fractured area below the unity RD/RS line.

Fig 17 demonstrates the resistivity ratio for fracture system identification. Many data points are located above the resistivity unity line (RD/RS = 1) with some clusters towards the massive featured hydrocarbon-bearing zone and more clusters towards the hydrocarbon-bearing fractures. Another significant cloud of data points is located below the unity line, indicating that the bulk of the basement reservoir is not fractured. For Lam and Meem members, no fracture systems exist, most probably because of their higher clay content (ductile minerals), suggesting other mechanisms for oil migration from these source rocks to the basement reservoir.

**Fig 18:**
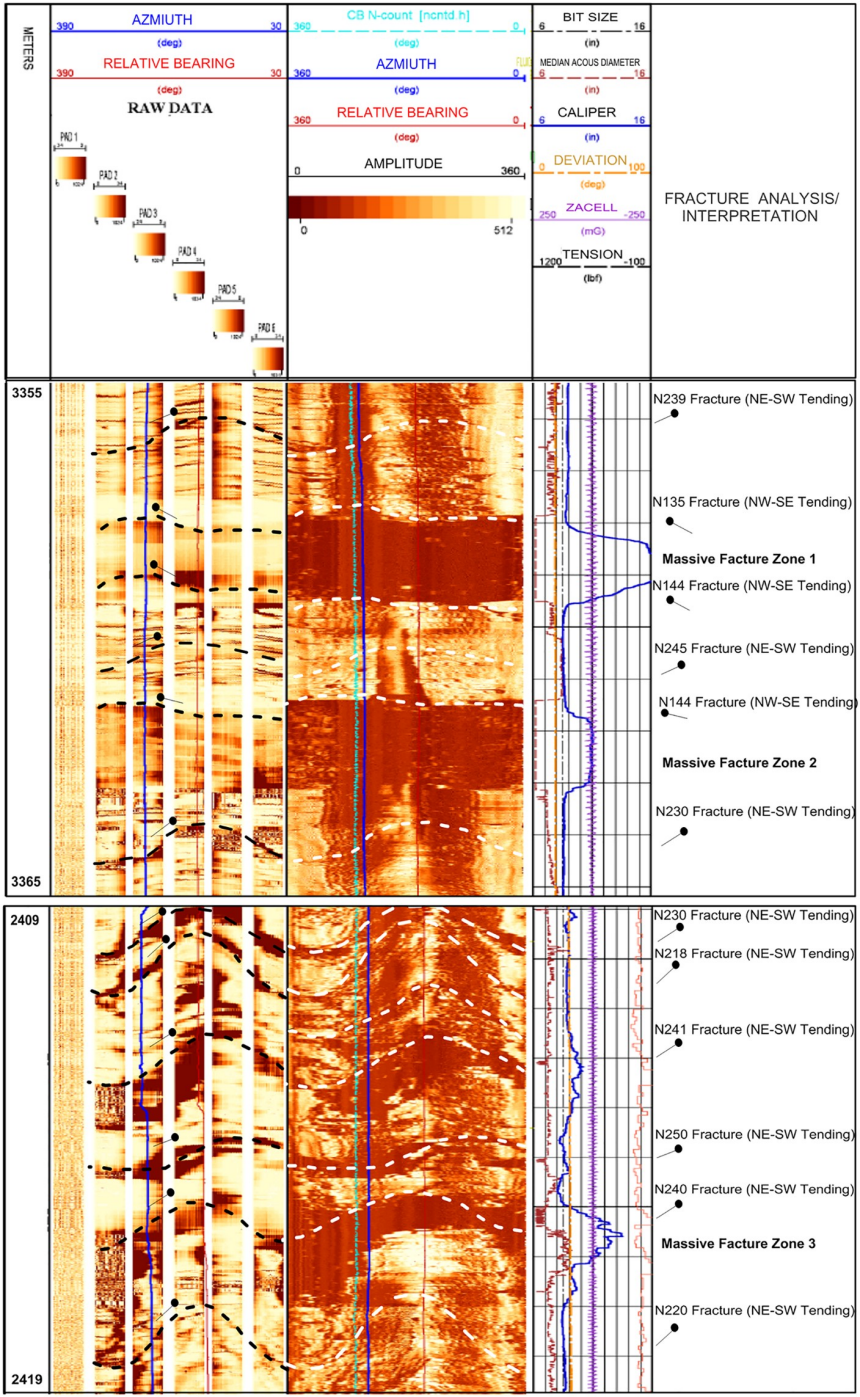
Interpretation of the image log of Habban-35 well. Fracture analysis and interpretation is indicated at the last track.

Fig 17a and 17b exhibits the interpretation of the image log that covers the reservoir intervals of 3355–3365 m and 2409–2419 m at the Habban-35 Well. Most fracture tadpoles dip mainly in the third (NE-SW) and second (NW-SE) quarters. The amplitude track shows a low dark-brown tone representing three massive open fracture zones associated with high caliper readings (the middle of Fig 18a for zones 1 and 2 and the lower part of Fig 18b for zone 3). Two of these massive fractures are oriented in the NW-SE direction (Fig 18a) and one is in the NE-SW direction (Fig 18b). The fracture system in the study area is fault-related rather than being of magmatic origin [16,45,46].

**Fig 19:**
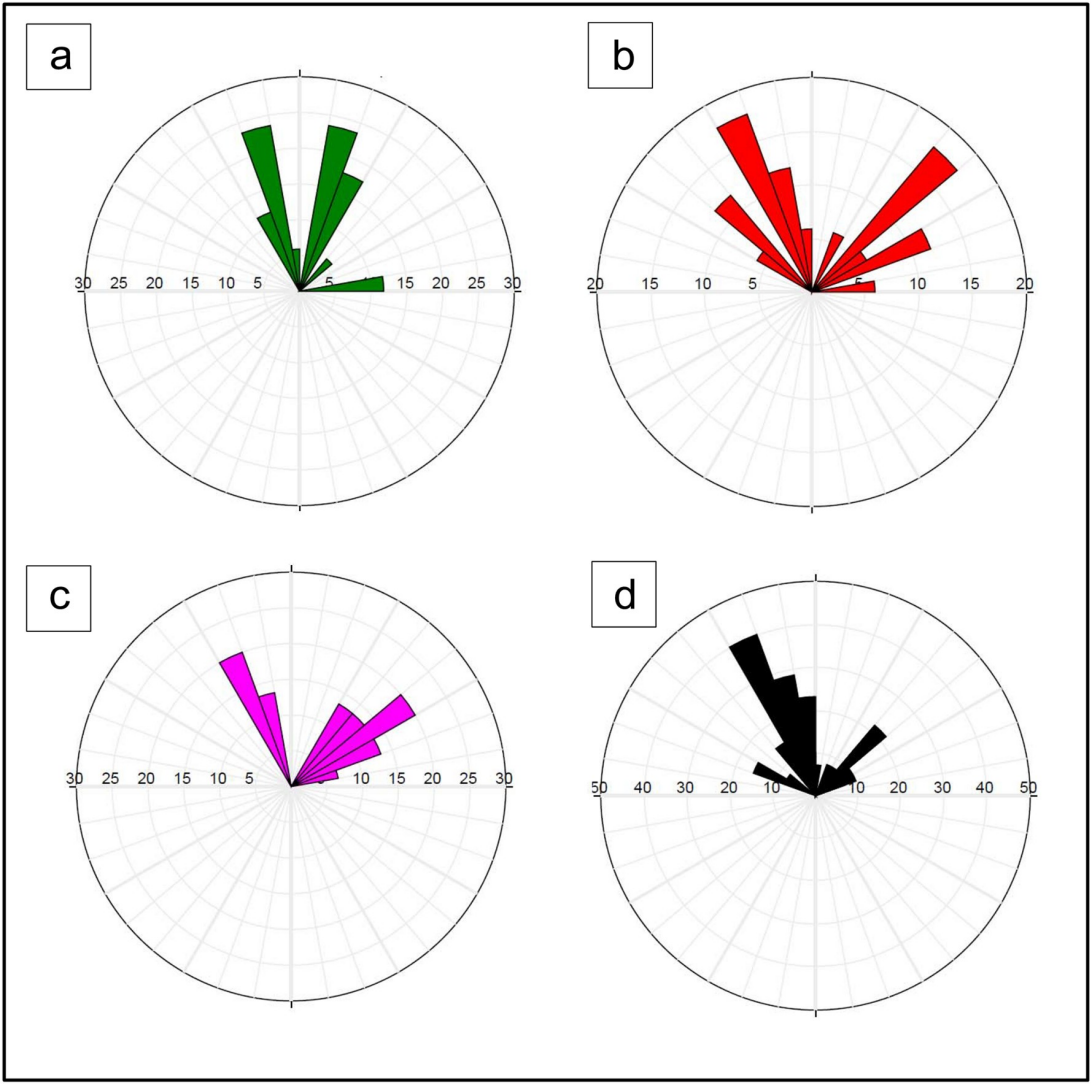
a) Rose diagrams for the fracture/fault system analysis detected from surface lineaments (outcrops), b) core plugs, c) FMI data, and d) interpretation of E-W seismic profiles.

The integration of fracture/fault system analysis from the surface lineaments (outcrops) and interpretation of subsurface data (image logs, core samples and seismic profiles) is enhanced through constructing number of rose diagrams (Fig 19).

Fig 19a and 19b highlights the facture/fault system analysis from the surface exposures and core samples, while Fig 19c and 19d shows rose diagrams for the results from FMI and E-W seismic data interpretation. Both of the NE-SW and NW-SE trending fracture/fault systems are well developed in all plots. However, some plots shows more intensity for NW-SE system (core and seismic, Fig 19b and 19d) than others. Overall, the fracture/fault system analyses based on the different approaches adopted here converge quite well.

### Fluid content and reservoir characteristics

**Fig 20:**
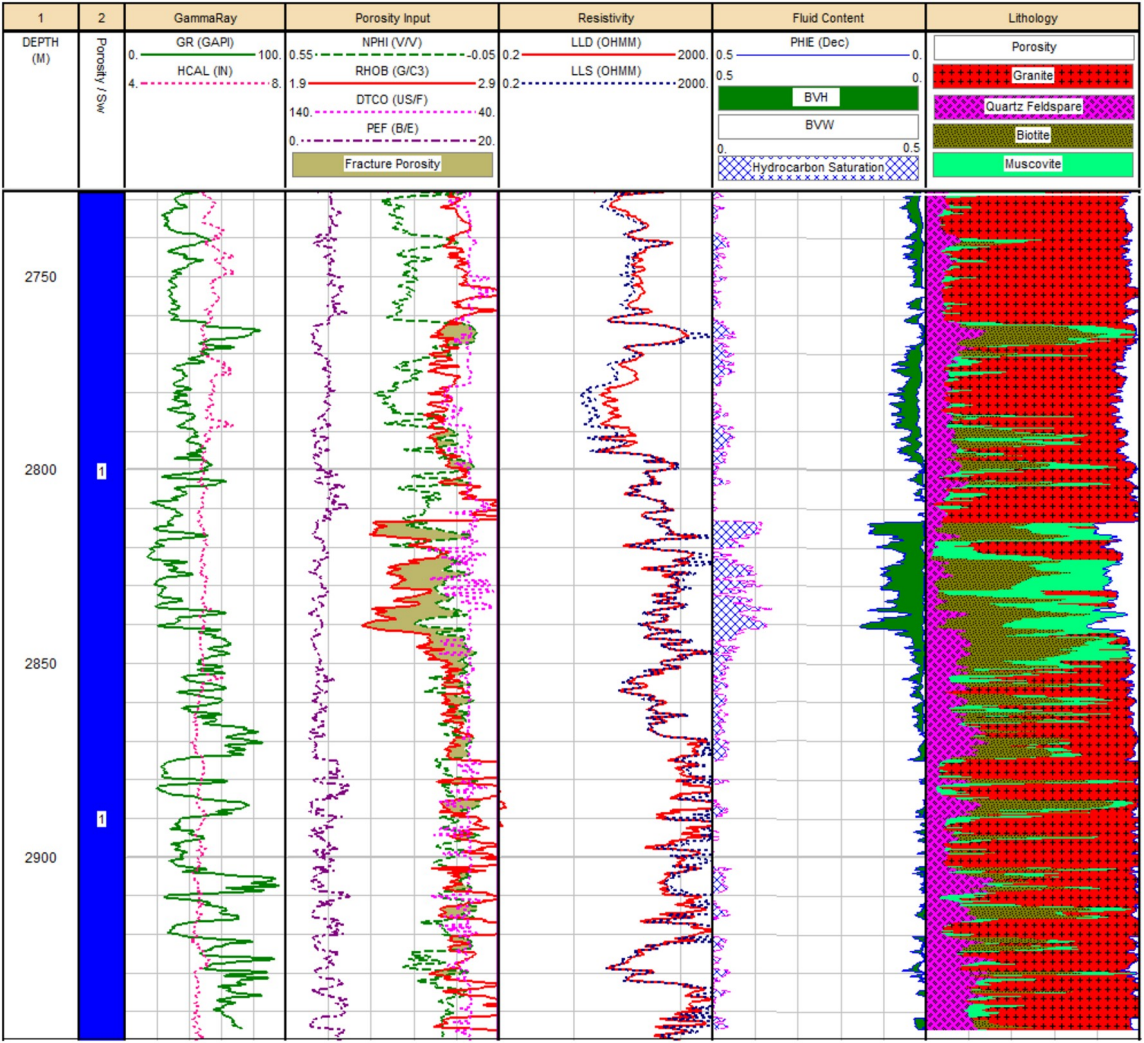
The petrophysical analysis of the basement reservoir of Habban-29 Well.

The petrophysical analysis of the basement reservoir of the Habban-29 Well is presented in Fig 20. Good hydrocarbon saturation is recognized in the middle and upper sections of the reservoir, more specifically in the front of the highly-fractured middle zone. This zone exhibits good fracture porosity up to 12% and dominant lithology of altered minerals (i.e., muscovite and biotite). The fracture porosity in the rest of the basement reservoir is less than 5% and the lithology is mainly granite and quartz-feldspar. Available oil samples from the Habban-1 Well, which has hydrocarbons at different zones of the fractured basement reservoir, were analyzed. Oil characteristics determined from the field measurements assigned 40° API @ 80 °F to the gravity of dead oil produced during the drill stem test. Measurements of separator gas samples showed a gas specific gravity of 0.8 (specific gravity of air is 1.0). H_2_S or CO_2_ was not detected. Downhole samples measured with an RCI had a gravity of 38.5 API at 60 °F.

### Seismic facies and hydrocarbon entrapment

The petroleum system in the Habban Field of the Sab’atayn Basin represents petroleum accumulation, generation, and migration from Jurassic source rocks into underlying basement rocks [29,30,49]. Basement reservoirs present good oil bearing characteristics in the Habban Field owing to extensively fractured and weathered uplifted blocks, which have been sourced by oil migrating through connected faults or fracture zones associated with basin evolution.

**Fig 21:**
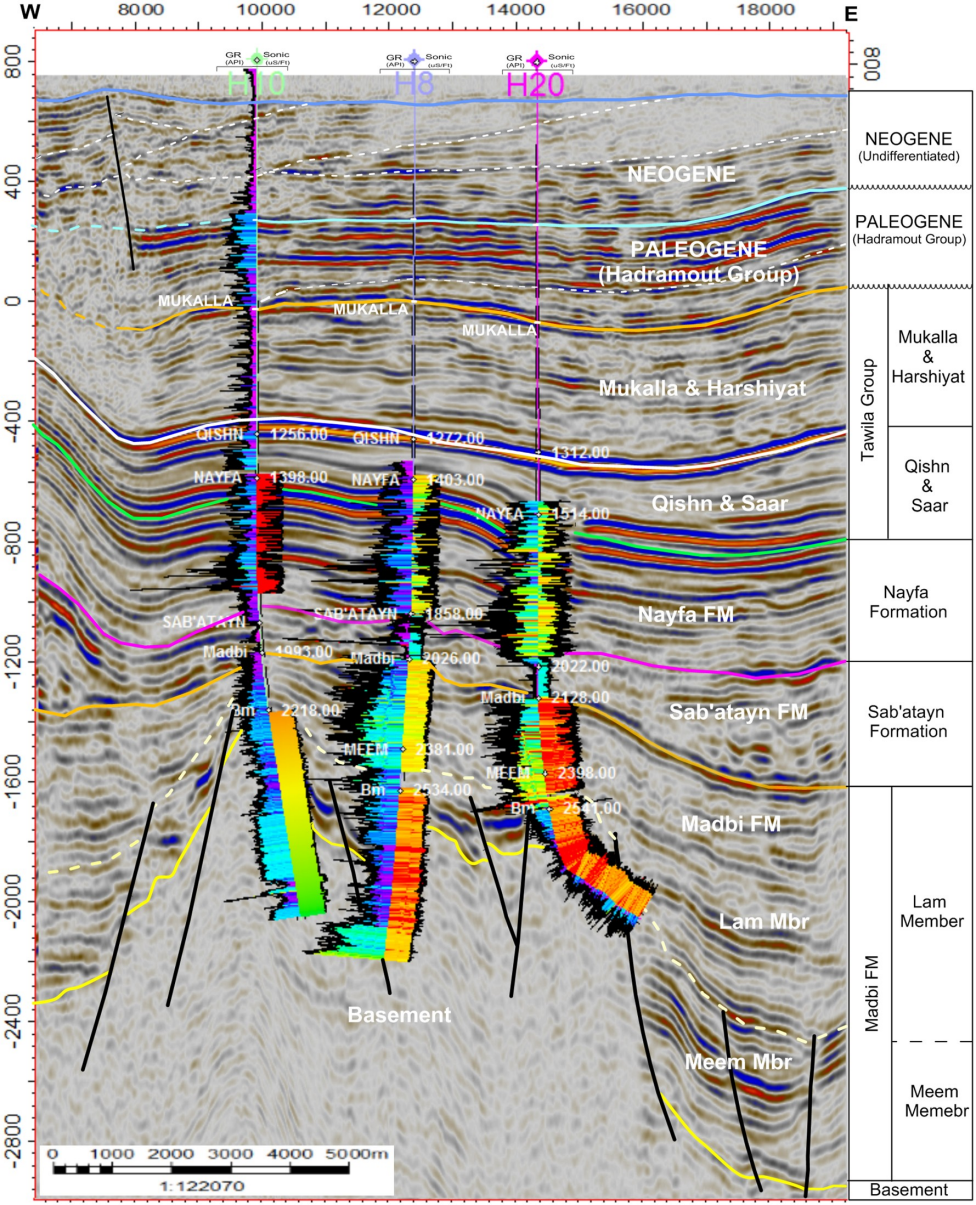
Seismic amplitude and facies analysis of inline seismic section 1335 (see Fig 2 for location). Gamma ray log (GR, left of wells) and sonic log (right of wells) are used for correlation. A column is provided with the names of the picked reflectors, to the right of the figure. The Paleogene and Neogene strata are undivided and unconformably overlying the Mukalla Formation of the Tawila Group. Tilting and convergent of reflectors is noticed at the Neogene sediments to the west of the seismic section.

**Fig 22:**
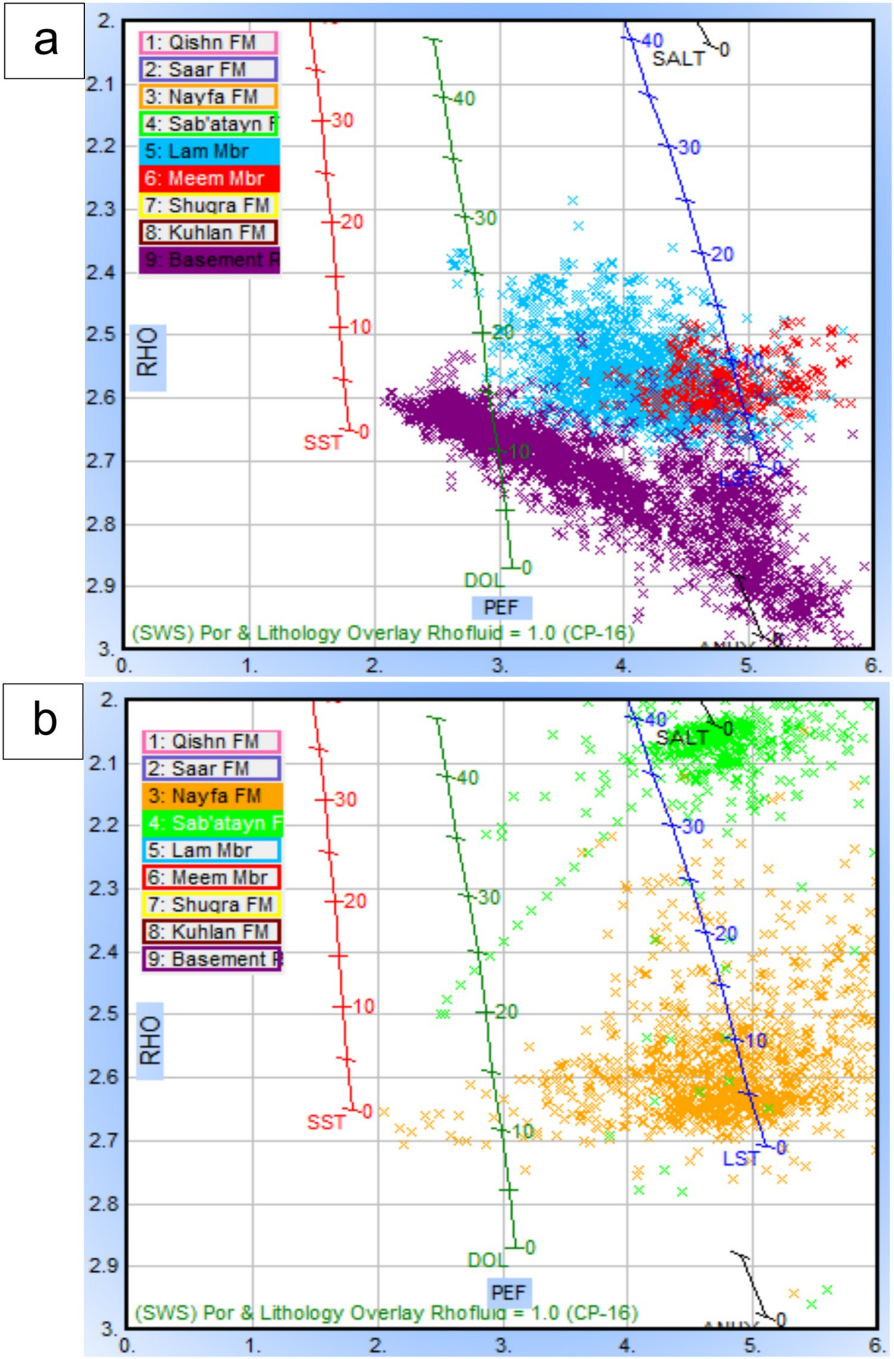
The photo electric absorption factor-density plot of Habban-8 well. a) A plot of the Lam Member (blue) and Meem Member. b) A plot for the Nayfa Formation (yellow) and Sab’atayn Formation (green).

The Lam Member of the Upper Jurassic Madbi Formation is a self-contained petroleum system because it acts as a good source rock for the underlying basement reservoir as well as a reservoir rock for some internally matured intervals [50–52]. Fig 21 shows the seismic amplitude and facies analysis of inline seismic section 1335 (see Fig 2 for location). Gamma ray log (GR, left side) and sonic log (right side) are used for correlation. The top of the LAM Member is picked at high gamma-ray log responses, which can be recognized during drilling based on the slow rate of penetration and the change in lithology from predominantly halite (Sab’atayn Formation) to an interbedded sequence of claystone, limestone, and sandstone. Strong seismic amplitude can be recognized by the dense limestone bed near the bottom of the Lam Member. The top of the Meem Member has a low seismic amplitude because claystone beds become thicker than the overlying Lam Member (Fig 21). However, the interbedded sandstone within the shale-dominated sequence of the Meem Member produces a bright package of fairly organized reflectors [28]. The mixed lithology of these members is indicated in the constructed Pe-RHO plot of the Habban-8 Well (Fig 22a).

The top of the Sab’atayn Formation exhibits a sudden change in velocity from the overlying high amplitude sequence of dense limestone and calcareous claystone of the Nayfa Formation to the underlying low reflectivity salt deposits. It is comprised of anhydrite and halite beds, associated with an increase in drilling rate and a decrease in the gamma-ray response (Fig 21). The evaporitic nature of the Sab’atayn Formation is quite clear (Fig 22b). The halite section is relatively thick preventing the overlain succession to accumulate hydrocarbons as it does in the Masilah Basin [28,53]. It offers a regional seal, together with the upper part of the Kimmeridgian Shale of the Madbi Formation (local seal), and often preserves high overpressure in the underlying formations and fractured basement reservoirs. In general, the Madbi and Sab’atayn formations have good thickness throughout the study area. The Qishn and Sa’ar formations is characterized by strong seismic amplitude at the top (carbonate bed) with a good overall overburden thickness that contributes to the underlying pressure [28,54]. A column is provided with the names of the picked reflectors, to the right of the figure. The Paleogene and Neogene strata are undivided and unconformably overlying the Mukalla Formation of the Tawila Group. Tilting of strata, termination and convergent seismic facies are noticed at the Neogene sediments to the west of the seismic section.

**Fig 23:**
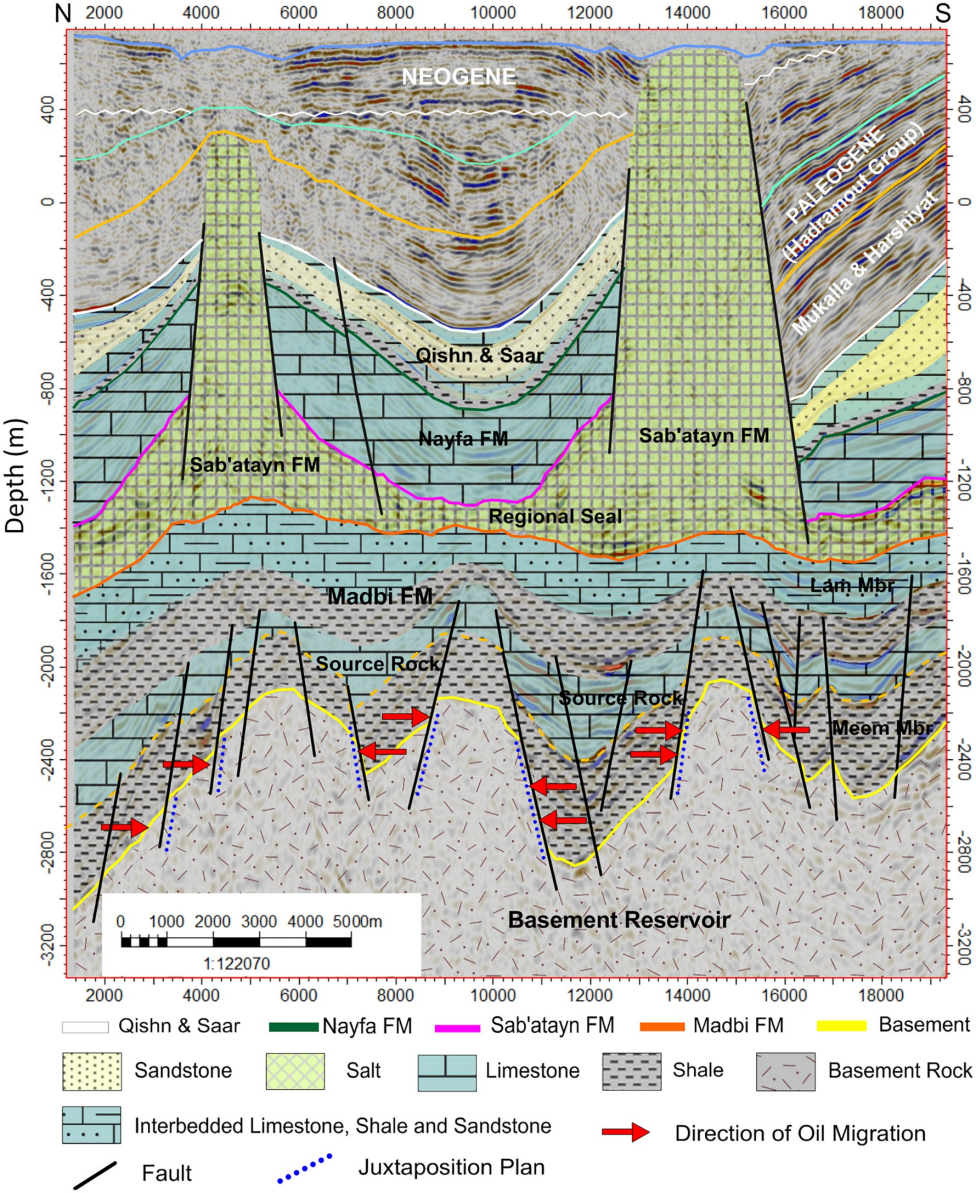
Geological cross-section inferred from the interpretation of inline seismic section. Directions of hydrocarbon migration from the source rock of Madbi Formation into the juxtapose-basement blocks (reservoir) along fault plans are indicated. The thick salts of the Sab’atayn Formation offered a good regional seal.

Oil migration into potential structural traps proceeded in an efficient manner because most structures were created before oil formed and migrated from younger Jurassic source rocks during tectonic movements. The mature hydrocarbon generation, expulsion, and accumulation started in the Late Cretaceous [50]. Charging took place by lateral or up-dip migration from the Late Jurassic Madbi Formation in nearby structural lows (Fig 23). Hydrocarbon emplacement was through fault juxtaposition of the fractured basement against the Late Jurassic organic shale source rock of the Madbi Formation into basement structural highs. Hydrocarbons are hosted in the basement horsts and overlain by seal formations.

## Conclusions

The Sab’atayn Basin is one of the two prolific Mesozoic basins located in central Yemen. It includes many hydrocarbon fields, among which the Habban Field, which produces oil from fractured basement reservoirs, is the most important. This study mainly focused on the characterization of the basement reservoir using 3D post-stack seismic data and well logs. The fracture system was identified from the outcrops, core samples, and borehole image logs. The main conclusions from this study are summarized as follows:

- Geologically, the Sab’atayn Basin is filled with Jurassic to Lower Cretaceous deposits and characterized by complex salt tectonics and faulting.
- The interpretation of 2D/3D seismic sections and depth structure maps reveals that the basement reservoir is primarily dissected by several NW-SE step-like normal faults (Najd Fault System) and, to a lesser extent, by NNE-SSW faults (Hadramauwt System).
- The Najd Fault System is more dominant than the Hadramauwt System, especially in the middle area where two prospective basement uplifts, Habban North High (HNH) and Habban Central High (HCH), are separated by a deep half-graben of Late Jurassic rifting. However, the northern and northeastern areas constitute the downthrown deep-seated structures of the basement reservoir.
- Fractures are clearly identified using the integrated logging plots and the resistivity ratio method. Most of the reservoir clusters are located within the hydrocarbon-bearing fractures, while a considerable amount of data are available for the massive featured hydrocarbon zones. Both the NW-SE and NNE-SSW fracture directions are clearly identified from the core, outcrop signatures, and image log analyses.
- Hydrocarbon saturation in the basement reservoir is associated with highly fractured zones in the upper and middle parts. Fracture porosity reaches 12%, while average reservoir porosity is less than 5%. The dominant lithology is granite, quantize/gneiss, quartz, and alkali feldspar with smaller amounts of altered of muscovite and biotite. Field measurements revealed the presence of crude oil with an API gravity of 40° and no H_2_S or CO_2_.
- A trap is created by the source characteristics of the overlying Sab’atayn and Madbi formations. The thick halite section of the Sab’atayn Formation offers a regional seal for the underlying basement reservoir. The Lam Member of the Upper Jurassic Madbi Formation is a self-contained petroleum system because it acts as a good source rock for the underlying basement reservoir beside being a reservoir rock.
- Hydrocarbon emplacement is through fault juxtaposition of the fractured basement blocks against the organic-rich shale of the Madbi Formation. Basement charging occurred through lateral or up-dip migration of hydrocarbons from the Madbi Formation in nearby structural lows.
- The uplifted basement reservoir blocks are of prime interest in terms of hydrocarbon accumulation and production. Most oil producing wells are drilled through the structural basement uplifts. Another uplift with good potential has been identified in the southeastern area for further hydrocarbon drilling/production activity.

## Supporting information

S1 Fig Uninterrupted 2D inline seismic section 1185, Happan filed, Yemen. (TIF)

S2 Fig Uninterrupted 2D inline seismic section 1235, Happan filed, Yemen. (TIF)

S3 Fig Uninterrupted 2D inline seismic section 1495, Happan filed, Yemen. (TIF)

S4 Fig Uninterrupted 2D inline seismic section 1695, Happan filed, Yemen. (TIF)

S5 Fig Uninterrupted 2D inline seismic section 1835, Happan filed, Yemen. (TIF)

S6 Fig Uninterrupted 2D crossline seismic section 221, Happan filed, Yemen. (TIF)

S7 Fig Uninterrupted 2D crossline seismic section 391, Happan filed, Yemen. (TIF)

S8 Fig Uninterrupted 2D crossline seismic section 581, Happan filed, Yemen. (TIF)

S9 Fig Uninterrupted 2D crossline seismic section 651, Happan filed, Yemen. (TIF)

S10 Fig Uninterrupted 2D crossline seismic section 741, Happan filed, Yemen. (TIF)

S11 Fig Uninterrupted 2D crossline seismic section 891, Happan filed, Yemen. (TIF)

S12 Fig Image log of Happan-35 well. (TIF)

S1 File Well logs of Happan-2A well. (LAS)

S2 File Well logs of Happan-24c well. (LAS)

S3 File Well logs of Happan-13 well. (LAS)

S1 Table Formation tops at Happan field. (XLSX)
