# Supplementary figures and images for: Characterization of a fractured basement reservoir using high-resolution 3D seismic and logging datasets: A case study of the Sab’atayn Basin, Yemen
Source: PLoS One. 2018 Oct 25;13(10):e0206079. doi: 10.1371/journal.pone.0206079 (PMC6201913; doi:10.1371/journal.pone.0206079)

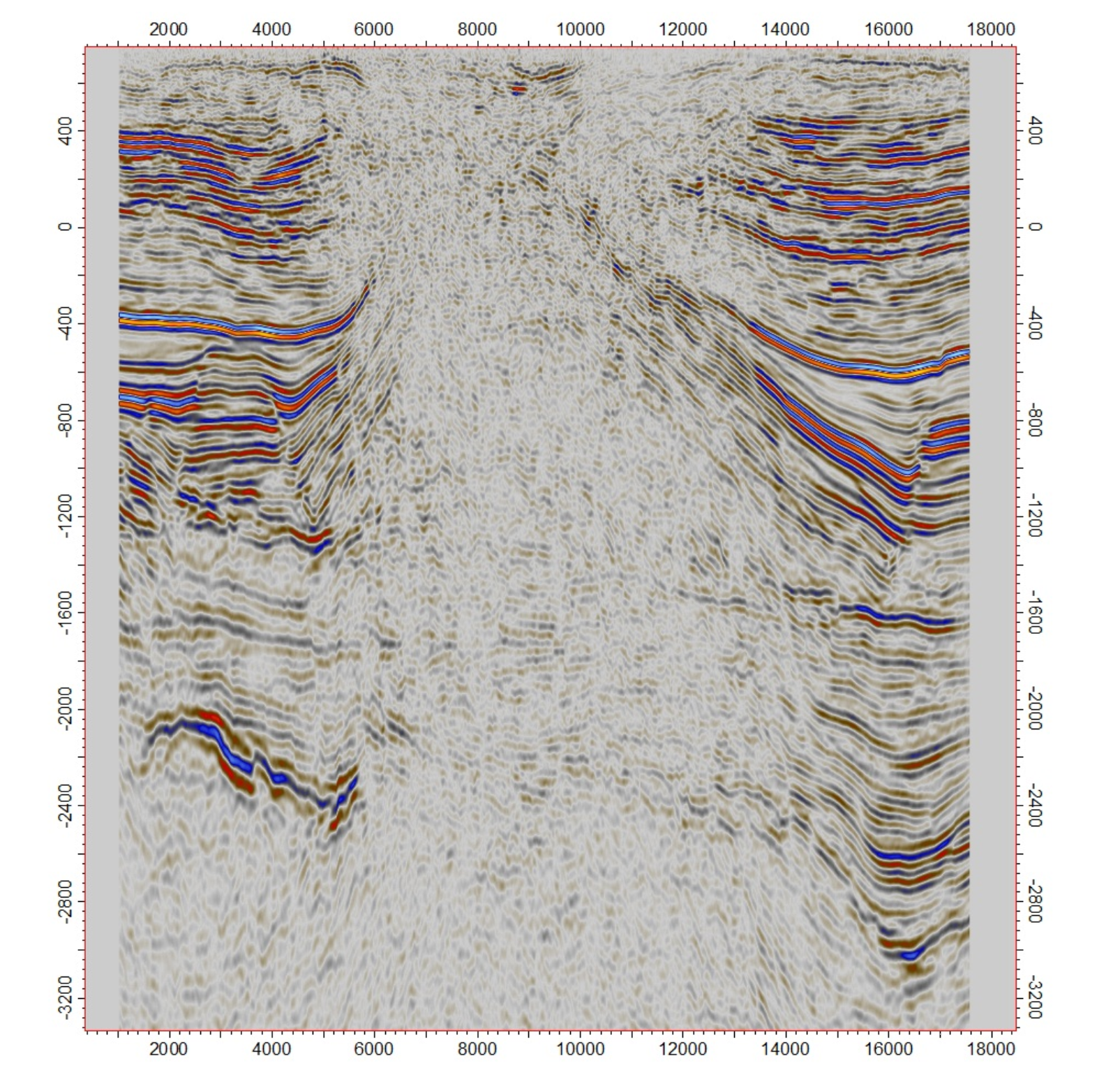

Supplement: S1 Fig — (TIF) [file pone.0206079.s001.tif]

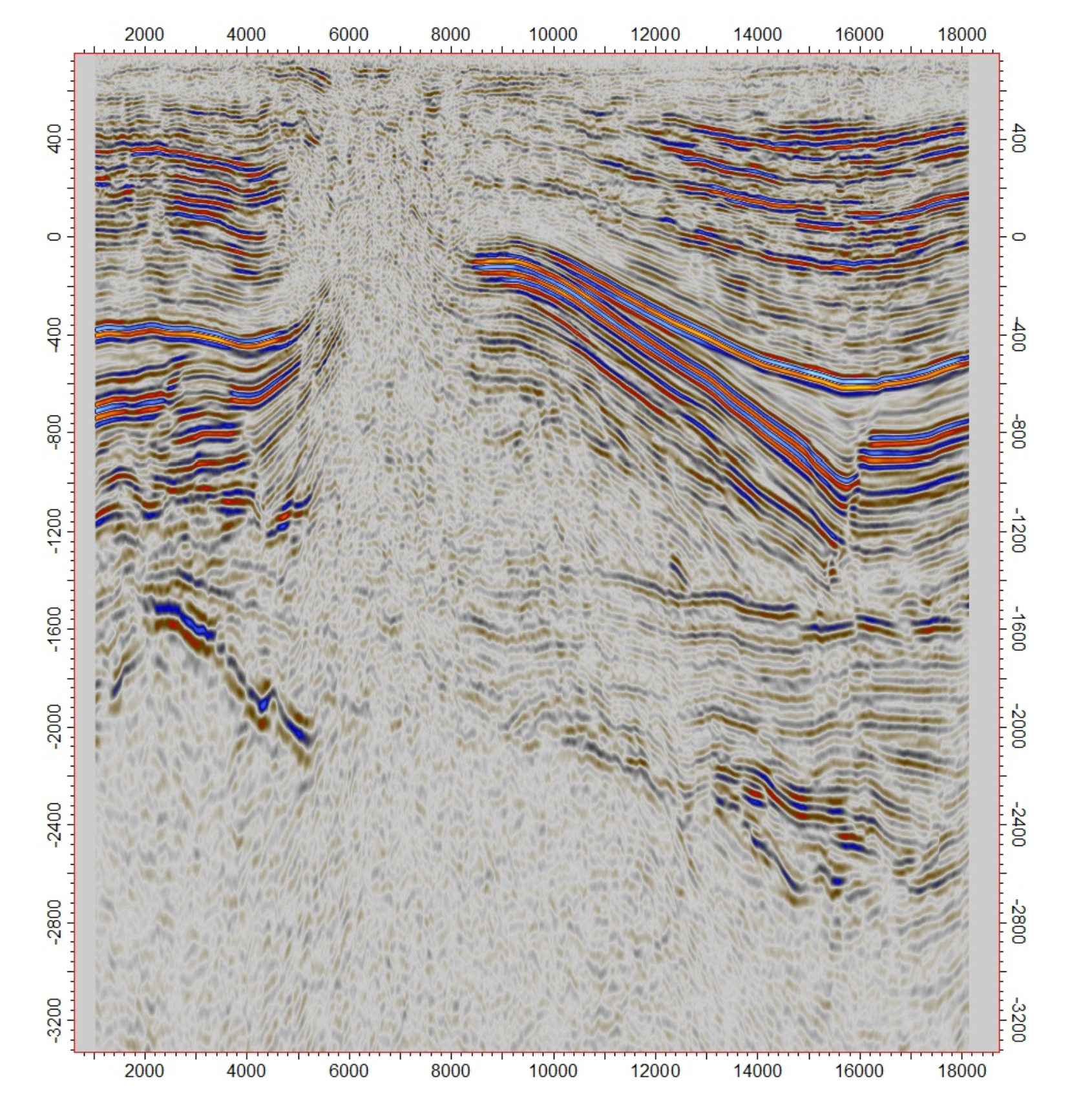

Supplement: S2 Fig — (TIF) [file pone.0206079.s002.tif]

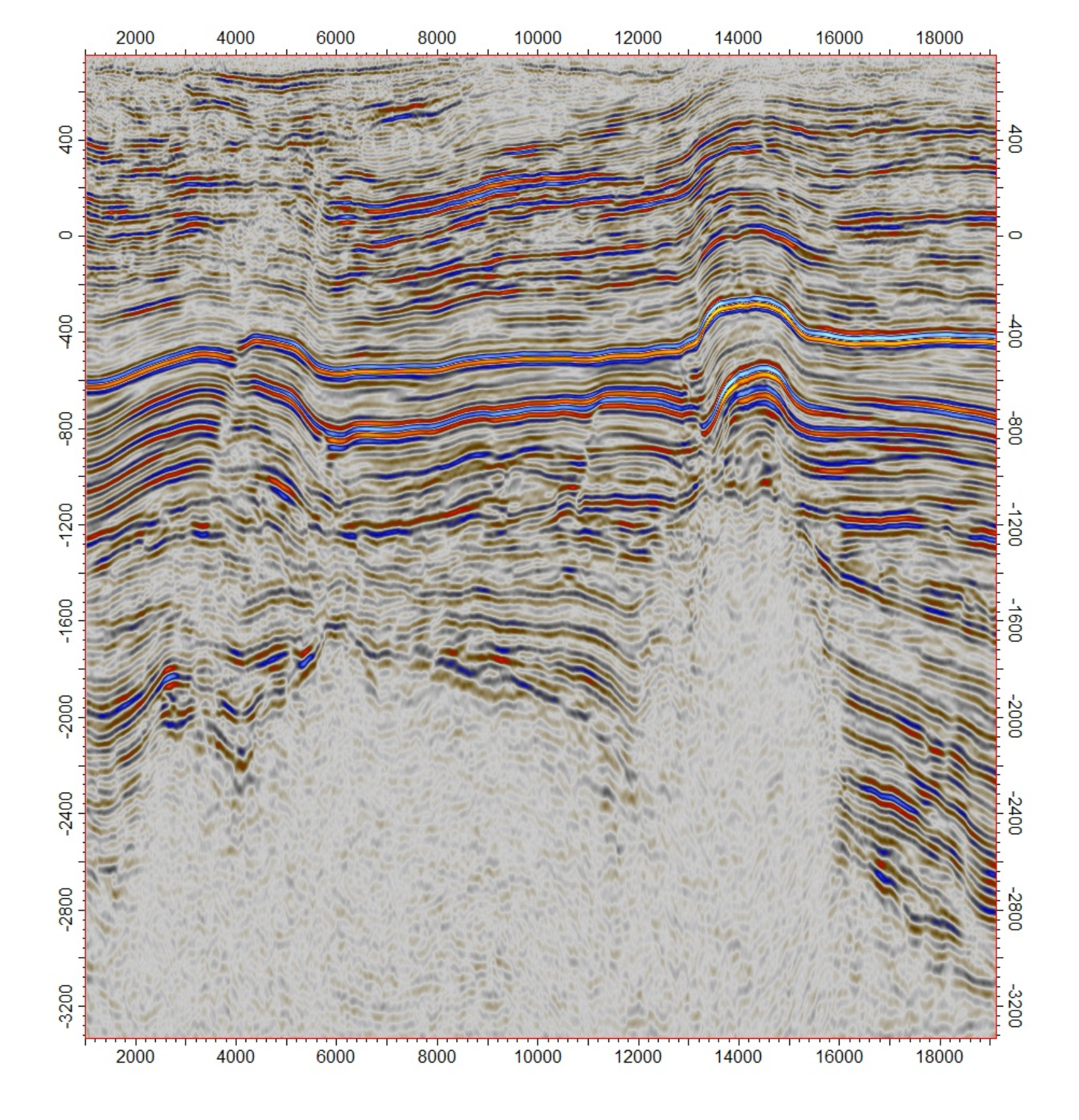

Supplement: S3 Fig — (TIF) [file pone.0206079.s003.tif]

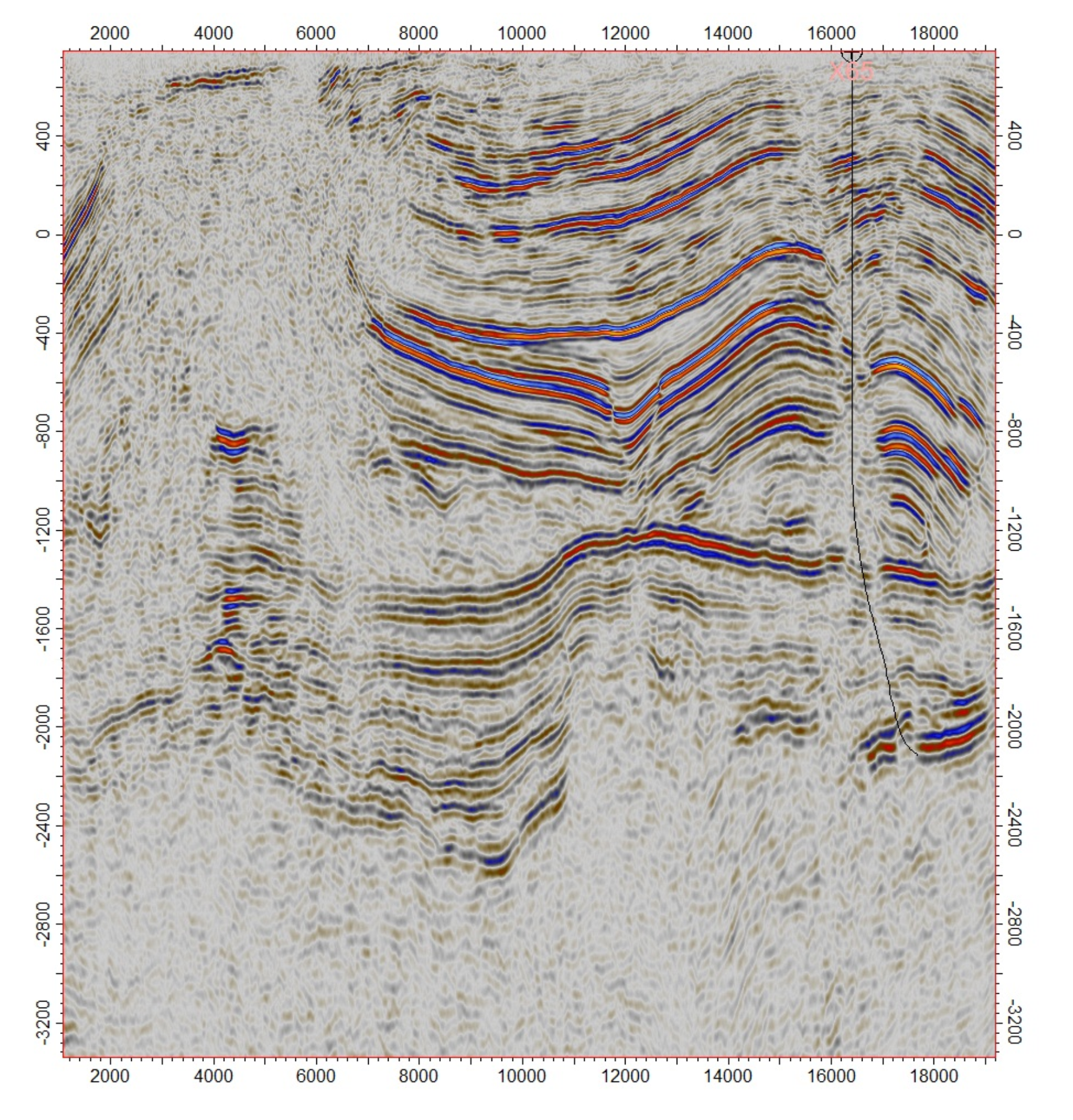

Supplement: S4 Fig — (TIF) [file pone.0206079.s004.tif]

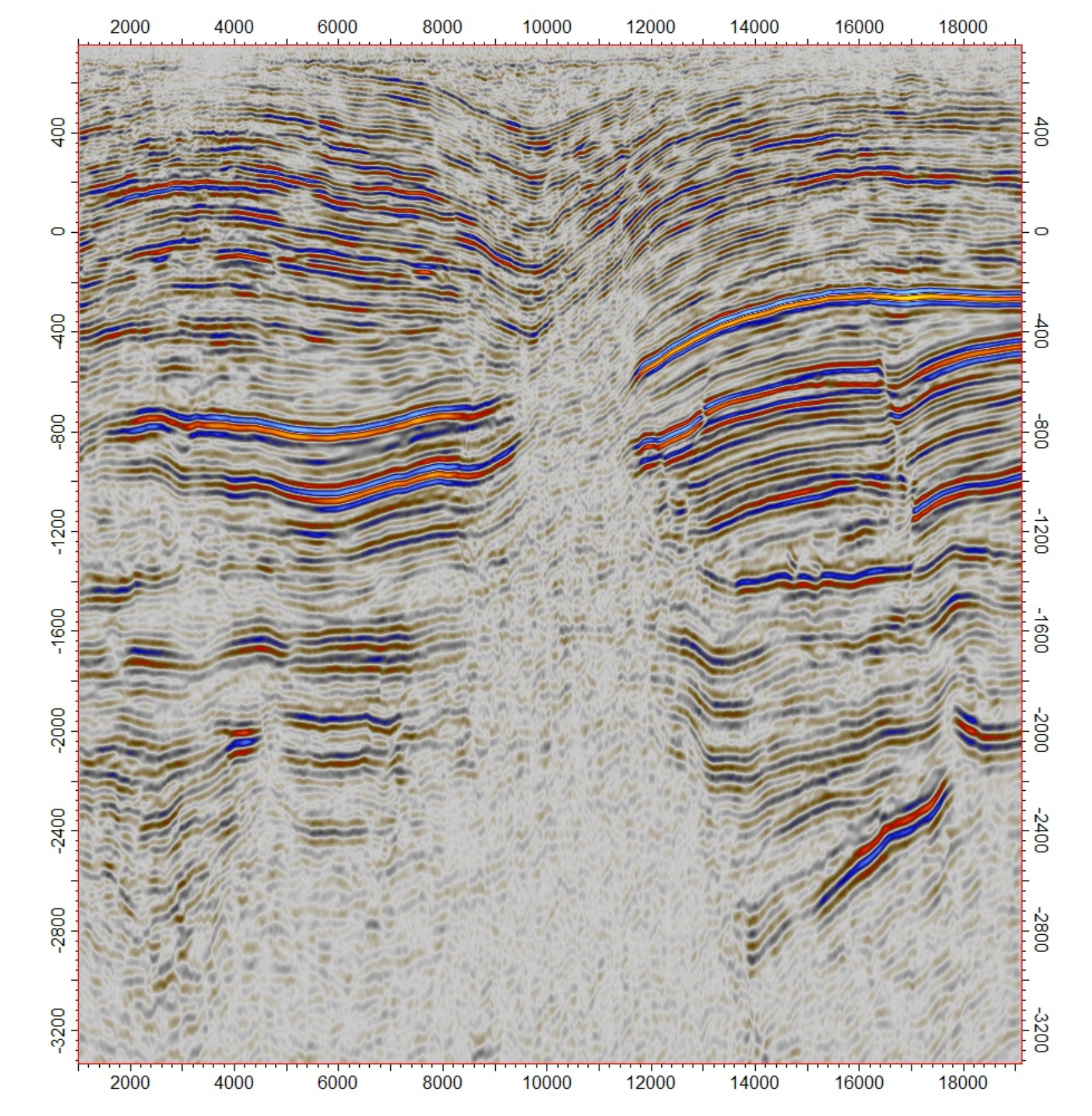

Supplement: S5 Fig — (TIF) [file pone.0206079.s005.tif]

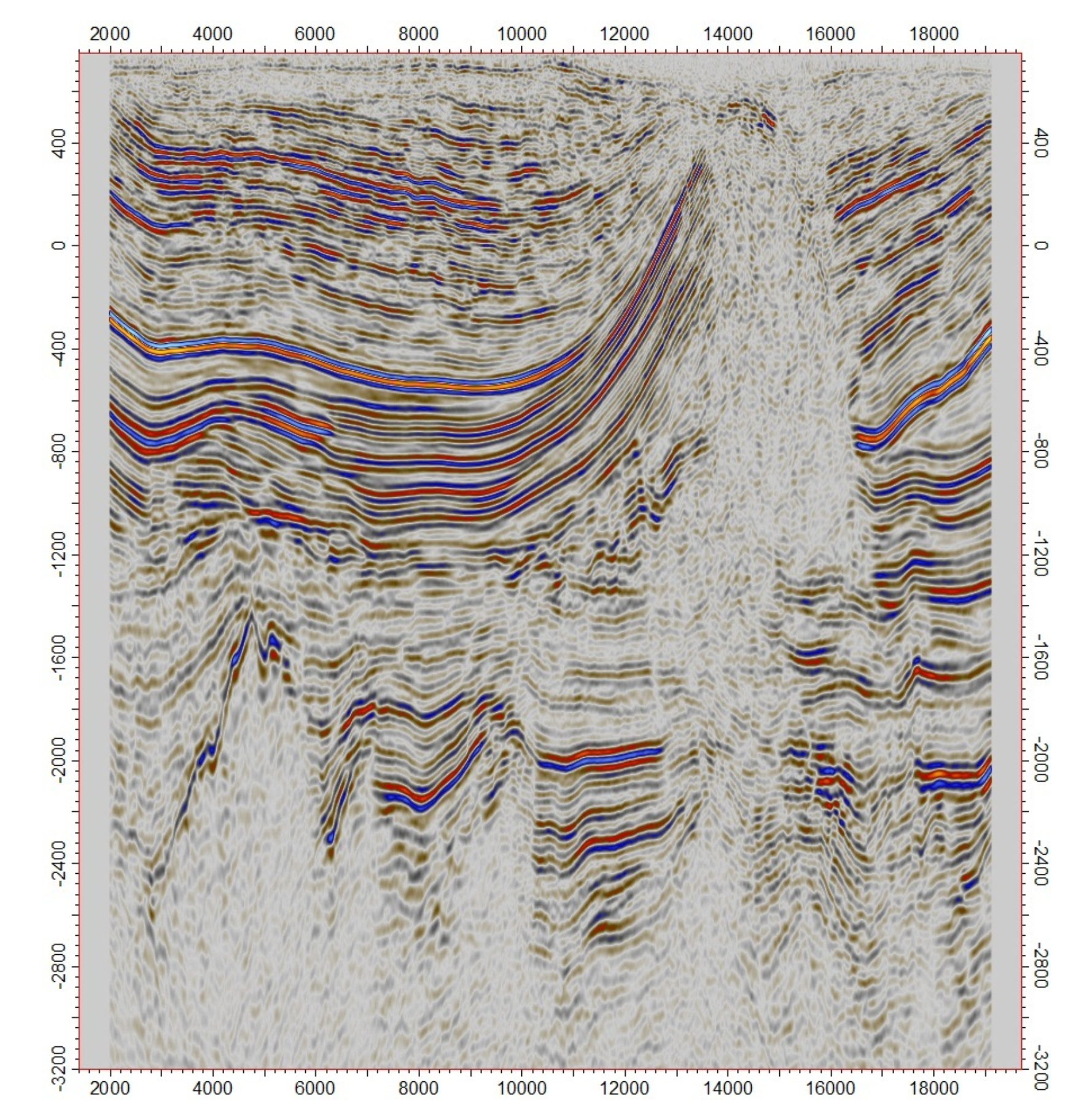

Supplement: S6 Fig — (TIF) [file pone.0206079.s006.tif]

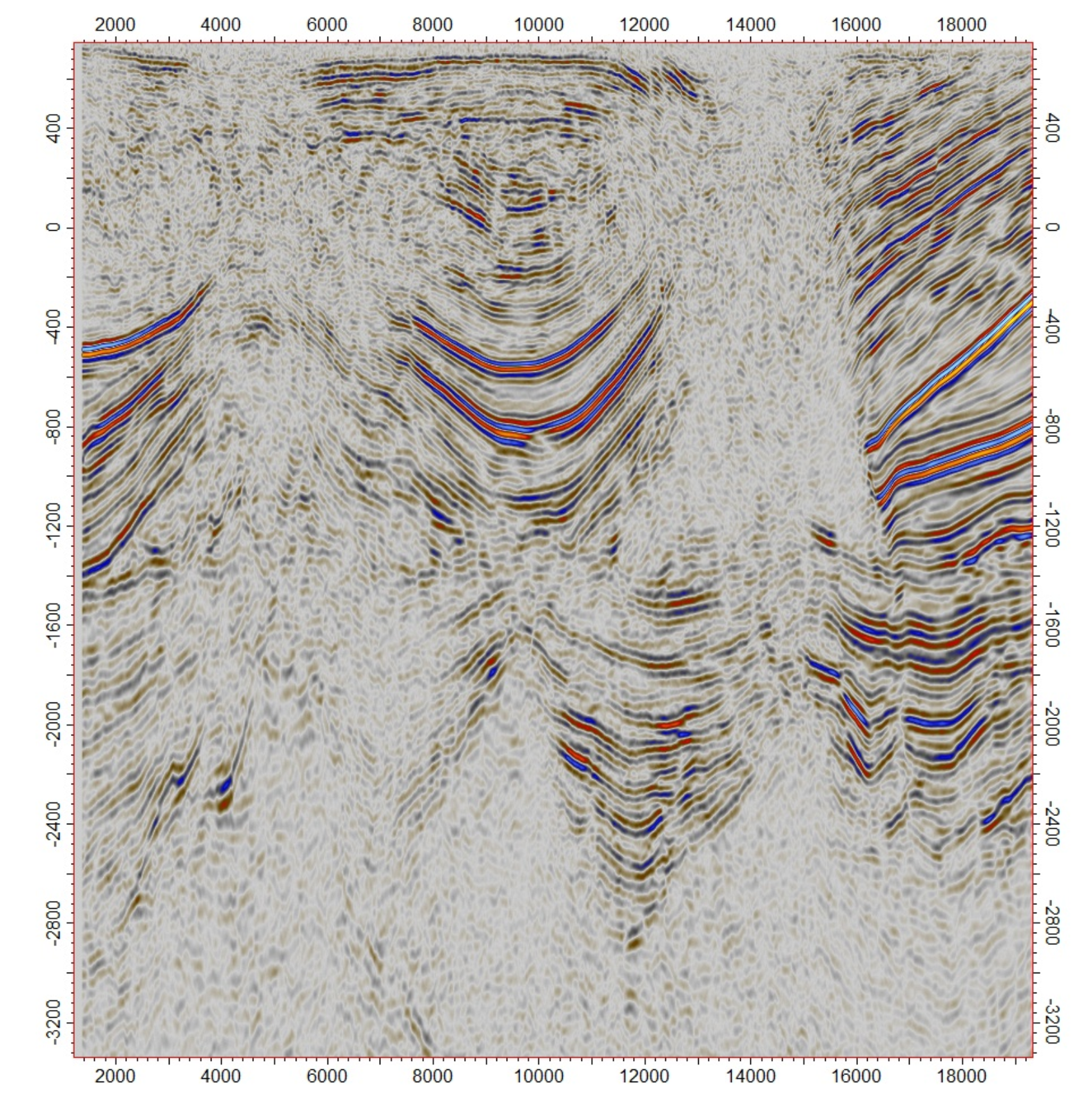

Supplement: S7 Fig — (TIF) [file pone.0206079.s007.tif]

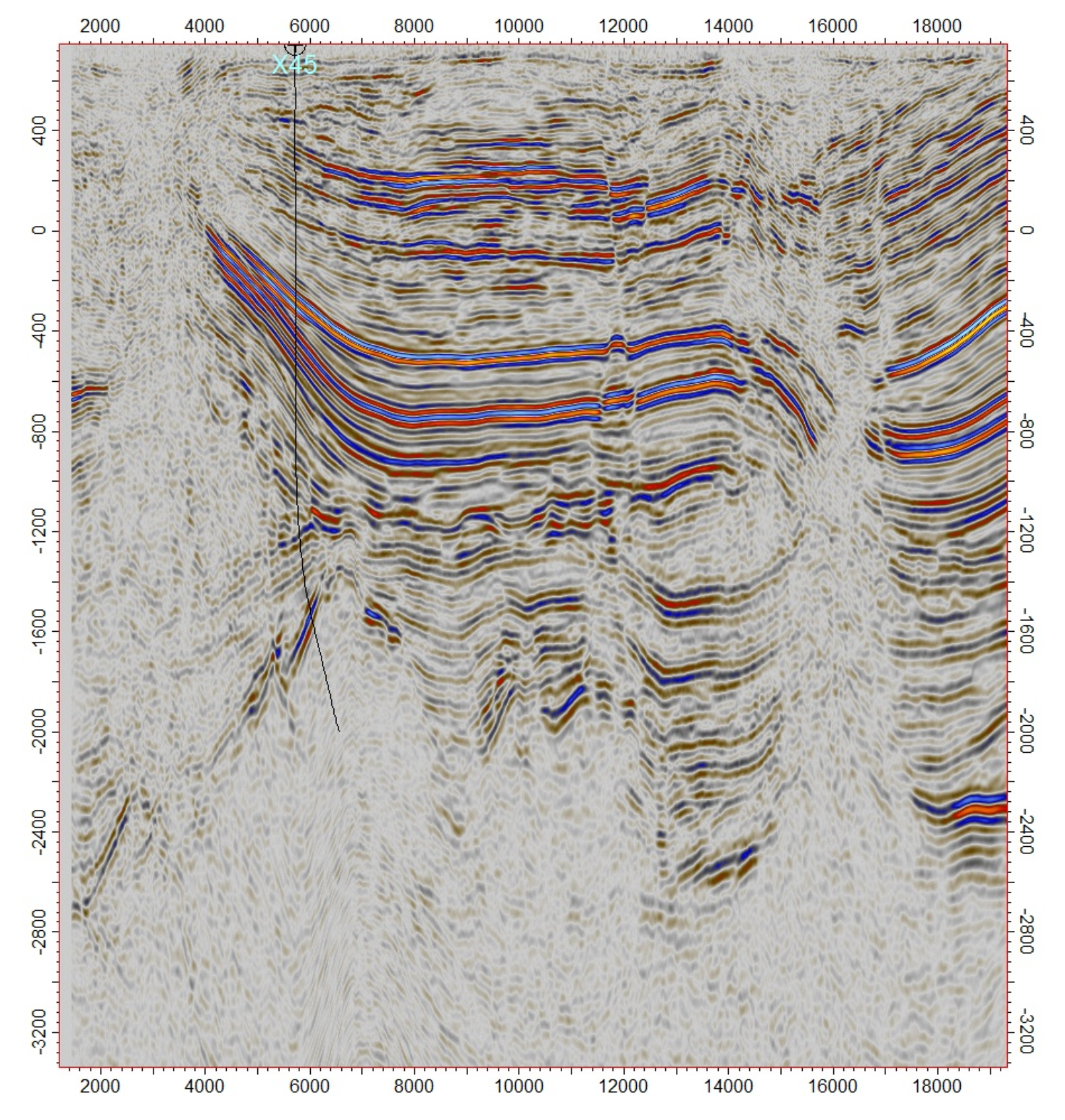

Supplement: S8 Fig — (TIF) [file pone.0206079.s008.tif]

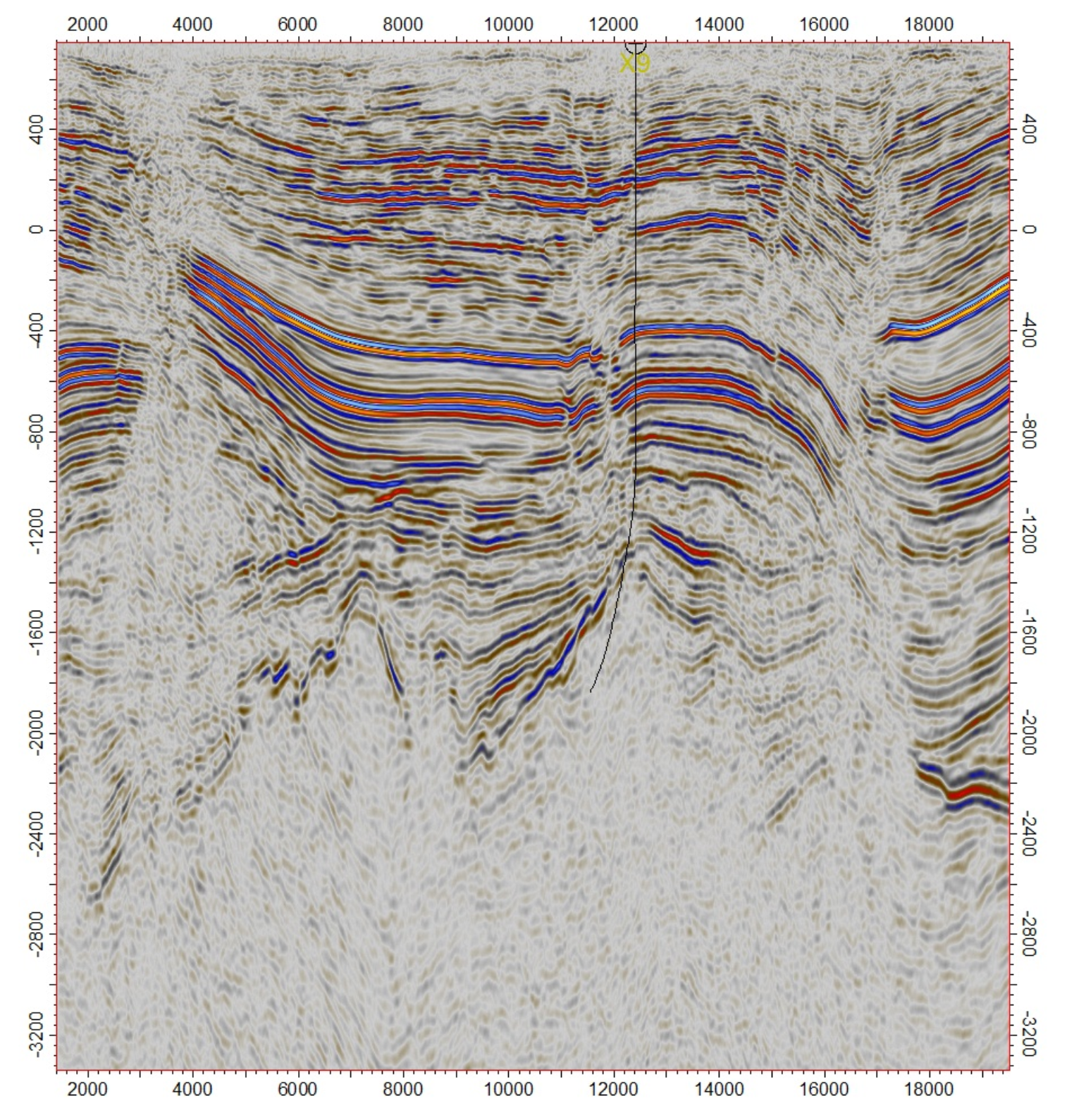

Supplement: S9 Fig — (TIF) [file pone.0206079.s009.tif]

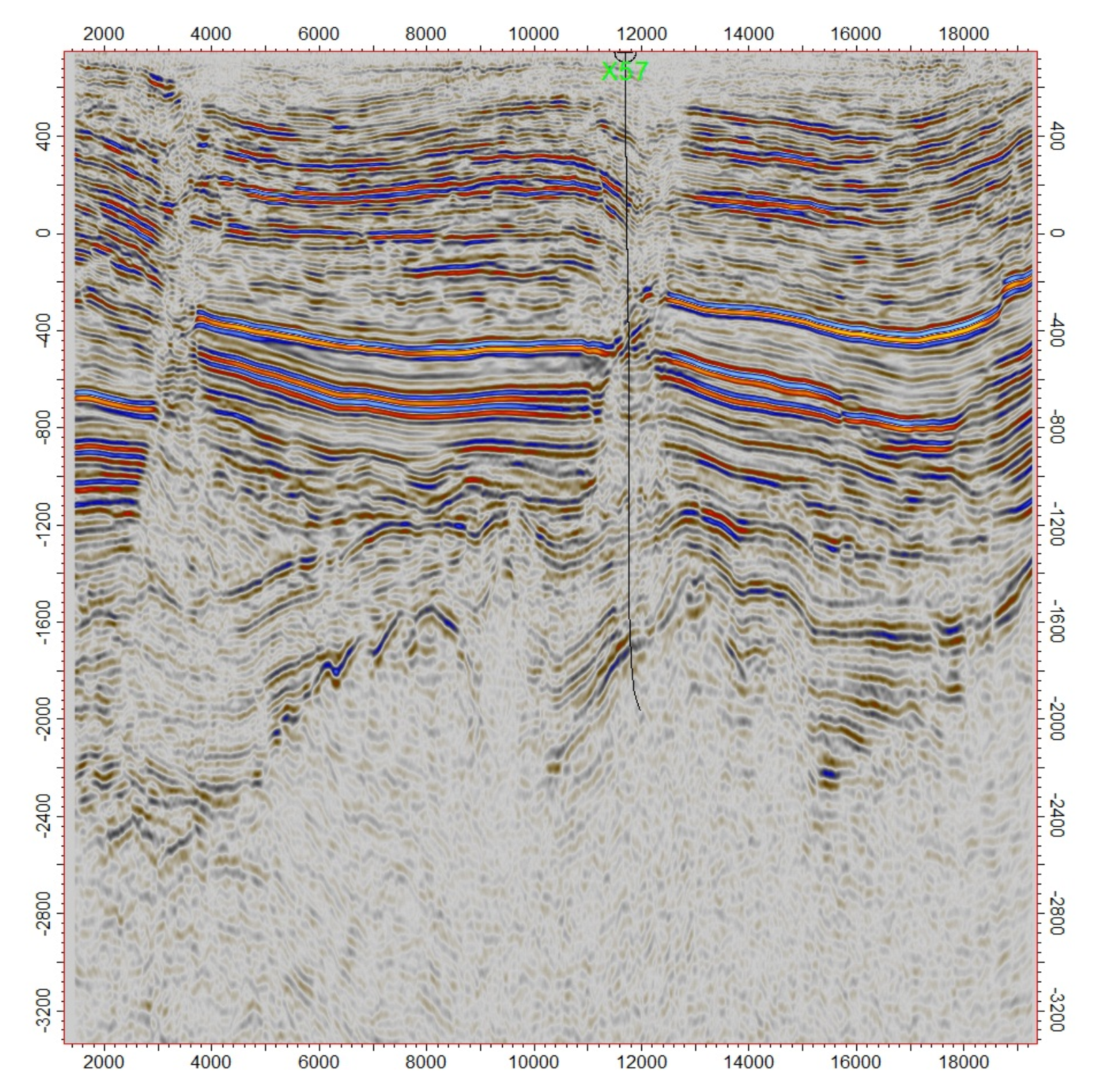

Supplement: S10 Fig — (TIF) [file pone.0206079.s010.tif]

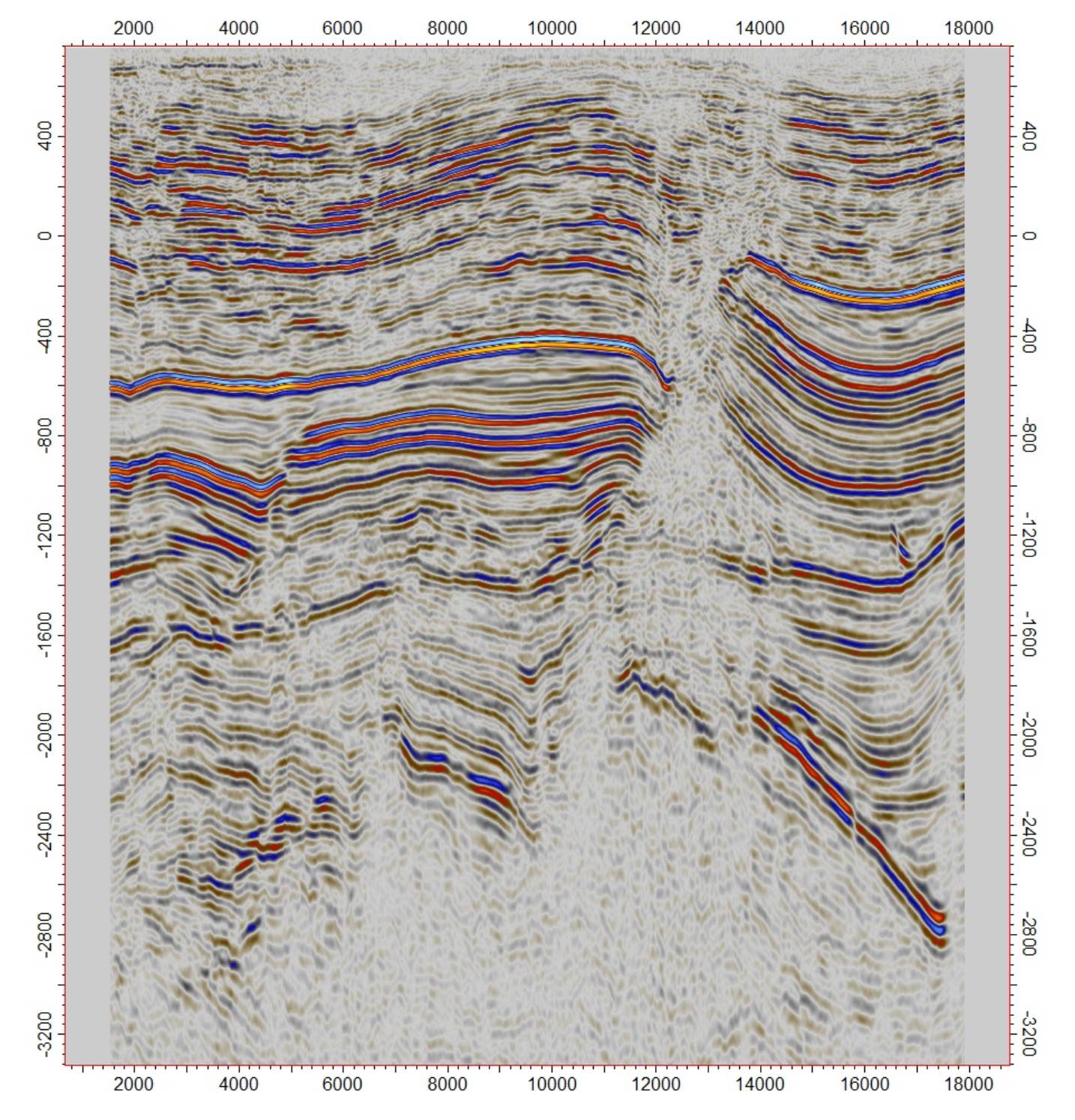

Supplement: S11 Fig — (TIF) [file pone.0206079.s011.tif]

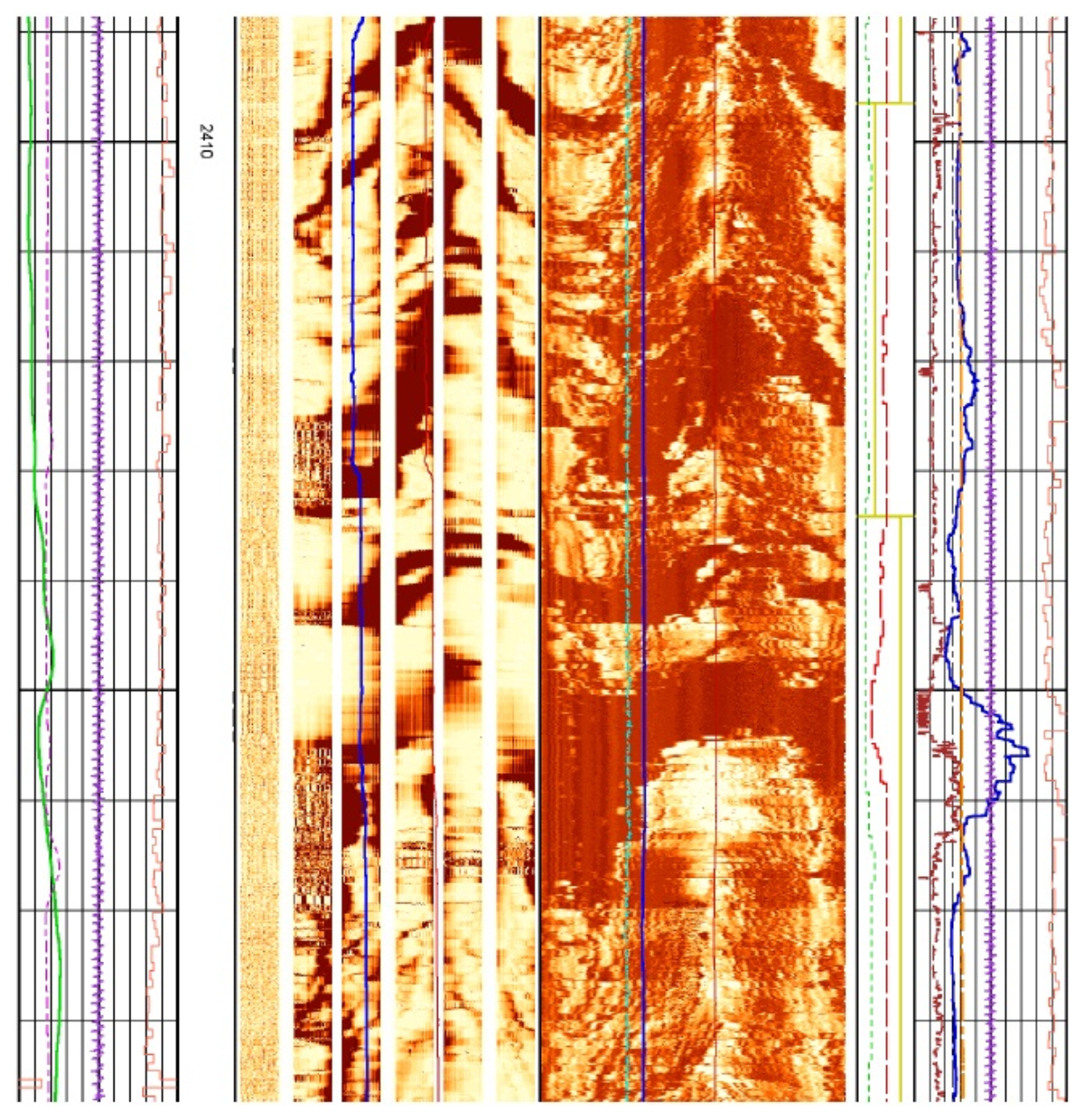

Supplement: S12 Fig — (TIF) [file pone.0206079.s012.tif]
